# A Method for Determining Incorporation Depth in Core–Shell UiO-66 Nanoparticles Synthesized Via Postsynthetic Exchange

**DOI:** 10.1021/acs.inorgchem.4c01787

**Published:** 2024-06-12

**Authors:** Adrian Hannebauer, Yaşar Krysiak, Andreas Schaate

**Affiliations:** † Institute of Inorganic Chemistry, Leibniz University Hannover, Callinstraße 9, 30167 Hannover, Germany; ‡ Laboratory of Nano and Quantum Engineering, Leibniz University Hannover, Schneiderberg 39, 30167 Hannover, Germany; § Cluster of Excellence PhoenixD (Photonics, Optics and Engineering—Innovation Across Disciplines), Leibniz University Hannover, Welfengarten 1A, 30167 Hannover, Germany

## Abstract

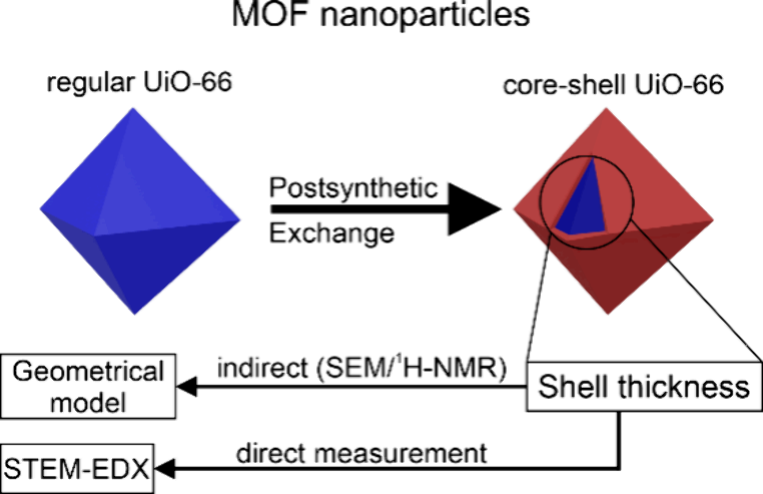

Postsynthetic exchange (PSE) is a key technique for integrating sensitive linkers into metal–organic frameworks (MOFs). Despite its importance, investigations into linker distributions have primarily focused on micrometer-sized crystals due to the analytical limitations, leaving nanoparticles less explored, although they are commonly synthesized and used in applications. In particular, the emergence of core–shell nanostructures via PSE has shown potential for applications in CO_2_ adsorption and selective catalysis. This study addresses this gap by investigating the formation of core–shell structures on nanoparticles under diffusion-controlled PSE conditions. By analyzing volume-to-surface ratios and conducting time-dependent experiments, we confirmed that these conditions facilitate the development of core–shell architectures. We also developed a straightforward method to calculate the minimum incorporation depth using basic parameters such as particle size and the total amount of incorporated linker. The accuracy of our approach was validated against data obtained from transmission electron microscopy coupled with energy-dispersive X-ray spectroscopy. These findings enhance the understanding of PSE in MOF nanoparticles and open up promising avenues for developing advanced MOF core–shell structures for various applications.

## Abstract 2

Our research investigates the core−shell structures in UiO-66 nanoparticles synthesized using diffusion-controlled postsynthetic exchange conditions. We introduce a method to determine the minimum incorporation depth of the exchanged linker. Confirmation of both the core−shell structure and the incorporation depth is directly achieved through scanning transmission electron microscopy coupled with energy-dispersive X-ray spectroscopy measurements.

## Introduction

Metal–organic frameworks (MOFs) are intriguing materials distinguished by their hybrid composition of inorganic building units interconnected by organic linker molecules. These structures are highly porous and offer customizable properties, facilitated by their modular nature and availability of diverse functional groups on the various linker molecules.^1^ While MOFs are typically synthesized via direct solvothermal methods, some structures or sensitive linkers require alternative methods such as postsynthetic modification (PSM).^2^ A key technique of PSM is postsynthetic exchange (PSE) or solvent-assisted linker exchange (SALE), which involves replacing an existing linker in the MOF framework with a new one, preserving the crystal structure.^3^ The mechanisms of the PSE process are complex and not fully understood, though recent research has begun to elucidate some aspects. Factors influencing PSE include a choice of solvent,^4−8^ reaction time,^4,8−11^ temperature,^9,10,12,13^ and the functional groups^12,14,15^ on the linker. The particle size of the original MOF used in the PSE process is also crucial and typically remains unchanged after PSE.^7−11,13,14,16,17^ Nair and co-workers demonstrated that, under identical reaction conditions, the incorporation rate for nanoparticles significantly increases compared to the micrometer-sized crystals.^10^ Additionally, particle size can change substantially after PSE, often due to a dissolution and recrystallization process that leads to a significant increase in the particle size.^18,19^ Furthermore, defects in the MOF structure significantly affect the exchange process.^4,8,12−14^ The incorporation of linkers at defect sites, referred to as postsynthetic linker insertion,^2^ competes with the process of linker exchange. The linker insertion is energetically favored and typically occurs prior to linker exchange.^4,8,12−15^

Recent research has primarily focused on understanding the linker distribution within the framework of MOFs, notably following the discovery of a core–shell structure of a MOF synthesized via PSE in 2017.^9,10^ It has been proposed that the distribution of linkers can be controlled by the kinetics of the PSE, which may be either diffusion- or reaction-controlled.^6^ In diffusion-controlled PSE, the linker exchange on the surface occurs more rapidly than diffusion into the core, leading to the formation of core–shell structures. Conversely, reaction-controlled PSE results in a homogeneous distribution of the linker, as the linker diffuses throughout the particle before the exchange takes place.

Various measurement techniques such as confocal laser scanning microscopy,^10,11,13^ confocal scanning Raman spectroscopy,^7−9^ Rutherford backscattering spectroscopy,^4,16^ nuclear magnetic resonance (NMR),^8,20^ and scanning electron microscopy coupled with energy-dispersive X-ray spectroscopy (SEM-EDX),^6,8,21^ have been employed to investigate linker distribution within MOFs. However, most of these methods face resolution limitations when applied to nanoparticles, necessitating the use of micrometer-sized MOF crystals. This presents a significant challenge, as micrometer-sized crystals are not readily synthesized and are limited in their applications.

Recent studies utilizing X-ray photoelectron spectroscopy (XPS) have provided insights into the linker distribution in MOF nanoparticles following PSE, confirming the presence of a core–shell structure.^17^ This was demonstrated by measuring the ratio of zirconium to iodine on the surface of the particle before and after ball milling—where the linker is expected to be uniformly distributed after ball milling. The higher iodine concentration observed before ball milling confirmed the presence of a core–shell structure. Despite these advancements, assessing the incorporation depth of the new linker within core–shell nanoparticles remains a challenging task.

Here, we introduce a straightforward method for calculating the minimum incorporation depth in MOF core–shell nanoparticles. We synthesized UiO-66 core–shell nanoparticles using diffusion-controlled PSE with H_2_BDC-Br and employed basic techniques such as SEM imaging and ^1^H NMR digestion experiments for this purpose. Our calculations are further validated by direct measurements of the incorporation depth using scanning transmission electron microscopy coupled with energy-dispersive X-ray spectroscopy (STEM-EDX). To the best of our knowledge, this is the first instance where the linker distribution of MOF core–shell nanoparticles synthesized via PSE has been measured, as a previous study measured the metal distribution of MOF-on-MOF structures.^22^

Our findings introduce an approach to calculate and directly measure the minimum incorporation depth providing valuable insights for future applications. MOF nanoparticles produced by PSE show potential in various applications, including gas separation,^23^ CO_2_ capture,^19,24^ water adsorption,^25^ and selective catalysis.^26^ With our method, the minimum incorporation depths of these MOF nanoparticles can now be readily determined, potentially linking incorporation depth to enhanced performances in these applications.

## Experimental Section

The detailed synthesis procedure and amounts are given in SI in Section 1. The synthesis procedures and characterization methods are summarized below. No uncommon hazards were noted.

### Synthesis of UiO-66 and UiO-66-Br Nanoparticles

UiO-66 and UiO-66-Br were synthesized in 100 mL Pyrex glass vessels by sequentially dissolving specific amounts of ZrCl_4_, demineralized water, formic acid (FA), and linker (H_2_BDC or H_2_BDC-Br) in *N*,*N*-dimethylformamide (DMF). The glass vessels were sealed and heated at 120 °C for 24 h. After cooling to room temperature, the precipitate was separated by centrifugation and washed once with DMF and twice with ethanol centrifuging after each washing step to remove the supernatant. The precipitate was then dried under a vacuum overnight. Finally, the obtained powders were purified by Soxhlet extraction with ethanol for 24 h and dried under a vacuum.

### Postsynthetic Ligand Exchange on UiO-66 and UiO-66-Br Nanoparticles

The postsynthetic ligand exchange was performed in 100 mL Pyrex glass vessels. For this specific amount of linker, (H_2_BDC or H_2_BDC-Br) was dissolved in 10 mL DMF at room temperature. After dissolution, specific amounts of MOF powder were added to the solution, and the vessels were sealed and heated at 120 °C for a certain time. After cooling to room temperature, the precipitate was separated by centrifugation and washed once with DMF and twice with ethanol centrifuging after each washing step to remove the supernatant. The precipitate was then dried under a vacuum overnight. Finally, the obtained powders were purified by Soxhlet extraction with ethanol for 24 h and dried under a vacuum.

### Characterization of MOF Nanoparticles and Exchanged MOF Nanoparticles

Powder X-ray diffraction (PXRD) was carried out in transmission mode using a Stoe Stadi-P diffractometer operated with Ge(111)-monochromatized CuKα_1_ radiation (λ = 1.54060 Å). A Mythen 1K detector was used, and the diffractograms were recorded from 2° to 50° 2θ and analyzed using the software *WinXPOW.*

The composition of the organic phase in the MOF powder samples was analyzed via liquid ^1^H NMR spectroscopy according to the procedure of Chu et al.^27^ For this purpose, 15 mg of MOF were digested in 1 M (NH_4_)_2_CO_3_ solution in D_2_O for 2 h. ^1^H NMR spectra were recorded on a Bruker Ascend 400 MHz Spectrometer and analyzed using the software *ACD-1D NMR processor.*

Argon physisorption measurements were performed at 87 K on a Micromeritics 3Flex instrument. The samples (roughly 25 mg) were activated at 120 °C under a secondary vacuum for 20 h. For the analysis of the data, the associated software *3Flex* was used. BET areas were determined by the BET-auto function of the software, and total pore volumes were calculated with the single-point method at a relative pressure of 0.95. The pore size distribution was determined using the “NLDFT—Argon on Oxides At 87 K” Kernel associated with the *3Flex* software using a regularization of 0.1.

SEM images were recorded with a Hitachi Regulus 8230 microscope using an accelerating voltage of 2 kV with a working distance of 7 mm. SEM samples were prepared by dispersion of small amounts of MOF powder in ethanol using an ultrasonic bath. The dispersion was dropped onto a polished graphite block and dried at room temperature. For the postproduction of SEM images, the software *ImageJ* was used.

The particle size and distribution were examined using dynamic light scattering (DLS). For this purpose, a 0.1 wt % solution of the MOF powder was prepared in ethanol and ultrasonicated for 30 min. For the measurement, 1 mL of the dispersion was transferred to a disposable cuvette cell. The measurements were performed on a Zetasizer Nano SZ from Malvern instruments and analyzed using the software *Zetasizer.* Every sample was measured three times, and the average of the measurements was used.

Transmission electron microscopy (TEM) images were performed on an FEI Tecnai G2 F20 TMP from FEI in scanning (STEM) mode at an acceleration voltage of 200 kV. The samples were dispersed in ethanol by ultrasonication and dropped on 400-mesh carbon-coated copper grids from Quantifoil and dried under air. STEM-EDX (energy dispersive X-ray spectroscopy) was performed by an EDAX Octane T Optima 60 SDD system.

## Results and Discussion

We begin by examining the effect of particle size on the exchange rate in PSE processes using UiO-66 nanoparticles of different sizes and quantifying the exchange rates through ^1^H NMR digestion experiments. This investigation sets the basis for further detailed analysis. Subsequently, we focus on the kinetics of the PSE, particularly comparing the rates of linker exchange to linker insertion. By conducting experiments on UiO-66 particles of uniform size, we isolate and examine the kinetic aspects of PSE without the effects of varying particle sizes. Finally, we introduce a method to determine the minimum incorporation depth of linkers in core–shell MOFs. We apply this technique to UiO-66 core–shell nanoparticles previously synthesized and validate the core–shell structure through proof of concept measurements using scanning transmission electron microscopy coupled with energy dispersive X-ray spectroscopy (STEM-EDX). This comprehensive analysis enhances our understanding of the PSE process and paves the way for more precise control over the synthesis and modification of MOF structures.

### Particle Size Influence on the Exchange Rate of PSE

**Figure 1:**
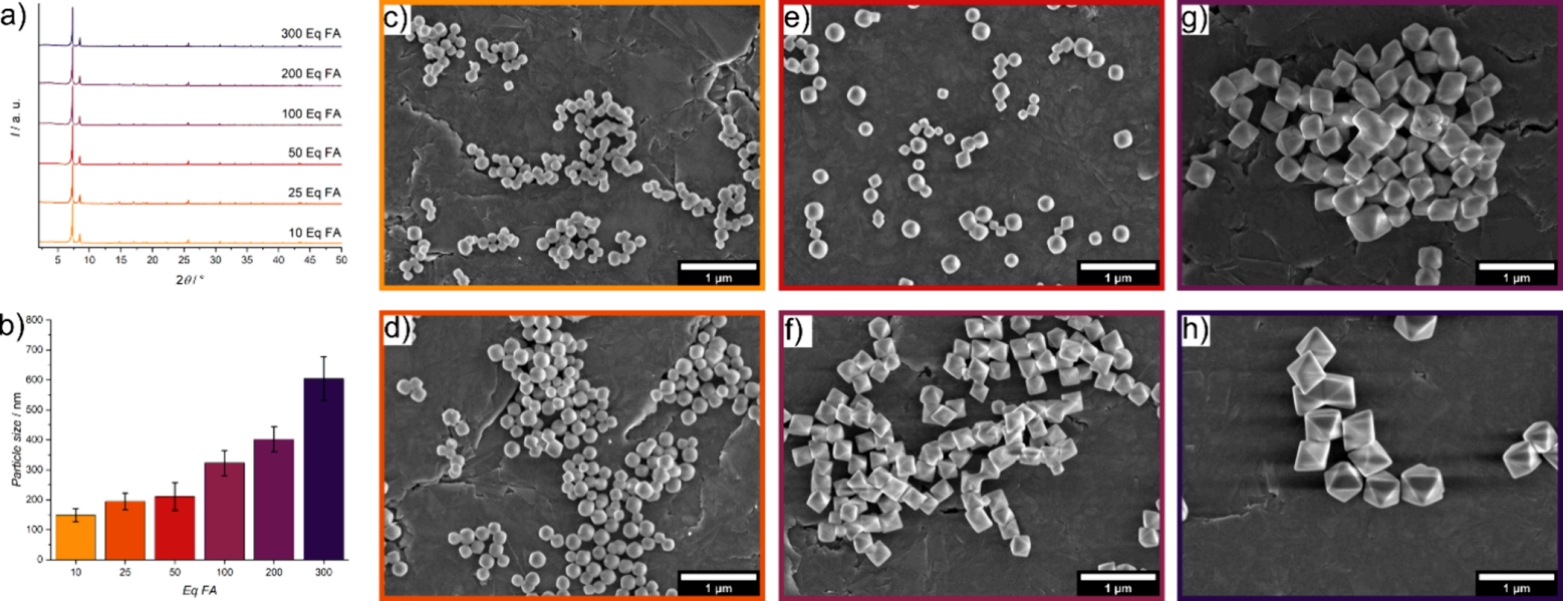
UiO-66 nanoparticles with increasing particle size were synthesized by increasing the equivalents (eq) of FA for PSE experiments. (a) PXRD measurements and (b) mean particle size UiO-66 nanoparticles measured from 100 particles from SEM images of (c) 10 eq FA, (d) 25 eq FA, (e) 50 eq FA, (f) 100 eq FA, (g) 200 eq FA, and (h) 300 eq FA used in the synthesis. The magnification of SEM images is 25000×.

We aimed to explore the minimum incorporation depth in UiO-66 nanoparticles across various sizes, emphasizing how particle size affects the exchange rate. This involved synthesizing six variants of UiO-66 nanoparticles using the established modulation method developed by our research group.^28^ By systematically increasing the amount of FA, we were able to control and enlarge particle sizes. Notably, the UiO-66 nanoparticles maintained high crystallinity, as shown in Figure 1a. We observed a clear increase in the mean particle size, from approximately 150 nm for the smallest particles (10 eq FA) to 600 nm for the largest (300 eq FA), as depicted in Figure 1b. The particle morphology also evolved from predominantly spherical to octahedral shapes with increasing amounts of modulator, as shown in SEM images (Figure 1c–h). This morphological transformation aligns with findings from our previous studies.^28^

We quantified the particle size of each sample from SEM images, analyzing 100 particles per sample. The resulting particle size distributions were narrow, characterized by small standard deviations (SD) and coefficients of variation (CV) (Figure S1). Additionally, we employed DLS measurements to assess particle size. While DLS confirmed the trend of larger UiO-66 nanoparticles with increased FA equivalents, it tended to overestimate the particle size compared to SEM measurements, due to its inclusion of the solvent shell in the measurement of the particle size (Figure S2). Despite the capability of DLS to analyze numerous particles simultaneously, we prioritized SEM for its superior accuracy in subsequent analyses.

Argon-sorption measurements on the UiO-66 nanoparticles confirmed the porosity with the BET-surface area increasing alongside the FA equivalents used in the synthesis (Figure S3). This increase can be attributed to a higher FA content within the framework, as verified by ^1^H NMR digestion experiments (Figure S4 and Table S1). The resulting higher defect concentration, due to FA incorporation instead of the linker, is reflected in the pore size distribution obtained from argon sorption measurements (Figure S3) and explains the occurrence of the 14 Å pore.^29^ The impact of these defects on the PSE will be explored further in the following chapter. Initially, however, our focus remains on the influence of the particle size.

**Figure 2:**
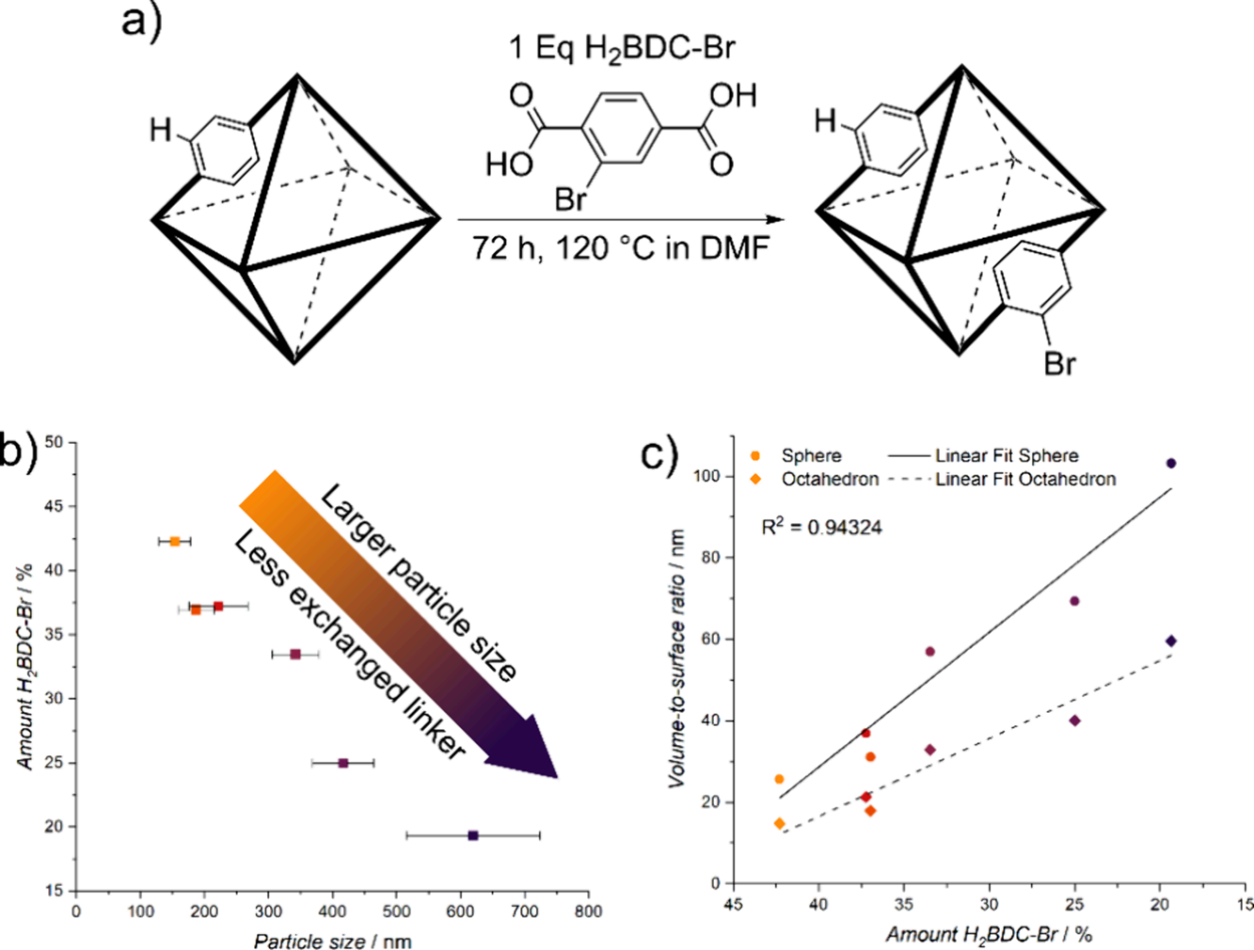
Overall linker exchange during PSE with schematic conditions of PSE shown in (a). A clear reduction in linker exchange is observed with increasing particle size of the UiO-66 nanoparticles (b), which can be attributed to the diffusion limitations inherent in the PSE process under these synthesis conditions. This is further demonstrated by the linear relationship between the volume-to-surface ratio and the amount of incorporated H_2_BDC-Br. (c) The volume-to-surface ratios are shown, assuming the particles have either a spherical (●) or octahedral (◆) shape.

To establish optimal PSE conditions, we consulted existing literature to synthesize core–shell nanoparticles via PSE. Targeting a high exchange rate within the shell, we chose DMF as the solvent because of its known capabilities in promoting the formation of core–shell structures with high exchange in the shell.^7^ We also utilized a low concentration of linker (1 eq relative to 1 eq of linker in the framework) to facilitate this architecture.^11^ Effective exchange in DMF for UiO-66 nanoparticles requires elevated temperatures,^12^ therefore, we conducted the reactions at 120 °C. Detailed information regarding the reaction conditions can be found in the SI in Section 1 while a schematic representation of the PSE process on the UiO-66 nanoparticle is illustrated in Figure 2a.

After PSE, all UiO-66 nanoparticle samples maintained their particle size, morphology, and crystallinity as confirmed through SEM, DLS, and PXRD measurements (refer to Figures S5–S10). Furthermore, we observed no surface etching or partial dissolution of the particles, phenomena that have been reported for ZIF particles under similar PSE reaction temperatures.^10,18^ Hence, we align with the existing literature in concluding that the PSE process on UiO-66 particles does not involve a dissolution and recrystallization process.^7−11,13,14,16,17^

We determined the amount of incorporated linker (H_2_BDC-Br) using ^1^H NMR digestion experiments (see Figure S12 and Table S2). The results demonstrate a significant correlation between the exchanged linker (H_2_BDC-Br) and the particle size of the UiO-66 nanoparticles (Figure 2b). In particular, the exchange rate decreases from over 40% for the smallest particles (ca. 150 nm) to about 20% for the largest particles (ca. 600 nm). We attribute this trend to the PSE conditions selected, which included a high reaction temperature and a low concentration of the new linker. Under these conditions, the exchange rate is primarily determined by the diffusion of the new linker into the framework.

In diffusion-controlled PSE, the amount of incorporated linker depends on the diffusion rate through the particle. Given that the diffusion rates are similar across all the UiO-66 nanoparticles due to uniform PSE conditions, the exchange process is primarily influenced by particle size and the corresponding volume-to-surface ratio. The difference in this measure is most pronounced when comparing nanoparticles to larger particles, with larger particles exhibiting a higher volume-to-surface ratio. We calculated these values for the UiO-66 nanoparticles, assuming spherical or octahedral shapes based on SEM images, and plotted it against the amount of incorporated linker (Figure 2c). The linear relationship observed strongly supports the notion of a diffusion-controlled process under the specified PSE conditions. These findings indicate that the UiO-66 nanoparticles likely exhibit a core–shell structure, aligning with previous observations for such nanoparticles.^17^

We conducted argon physisorption measurements to assess the porosity of the UiO-66 nanoparticles after the PSE (refer to Figures S13–S15). Despite remaining highly porous, the nanoparticles exhibited a linear decrease in BET-surface area and pore volume with increasing amounts of exchanged linker. This reduction can be ascribed to the substitution of small hydrogen atoms with larger bromo groups in the newly incorporated linker. Additionally, changes in the pore size distribution were observed after PSE, including a shift of the 14 Å pore to smaller diameters (12 Å) and a decrease in intensity. This shift can be attributed to the insertion of linkers into defects previously capped by FA, as confirmed by ^1^H NMR digestion experiments (see Figure S12 and Table S2). Notably, particle size had a more pronounced effect on the exchange rate than defect concentration. Larger particles, despite having a higher concentration of FA and consequently more defects, exhibited less linker exchange. Therefore, to minimize the impact of particle size variability, we conducted our PSE investigations on the kinetics using particles of uniform size.

### Kinetic Investigation of Linker Insertion Versus Linker Exchange

**Figure 3:**
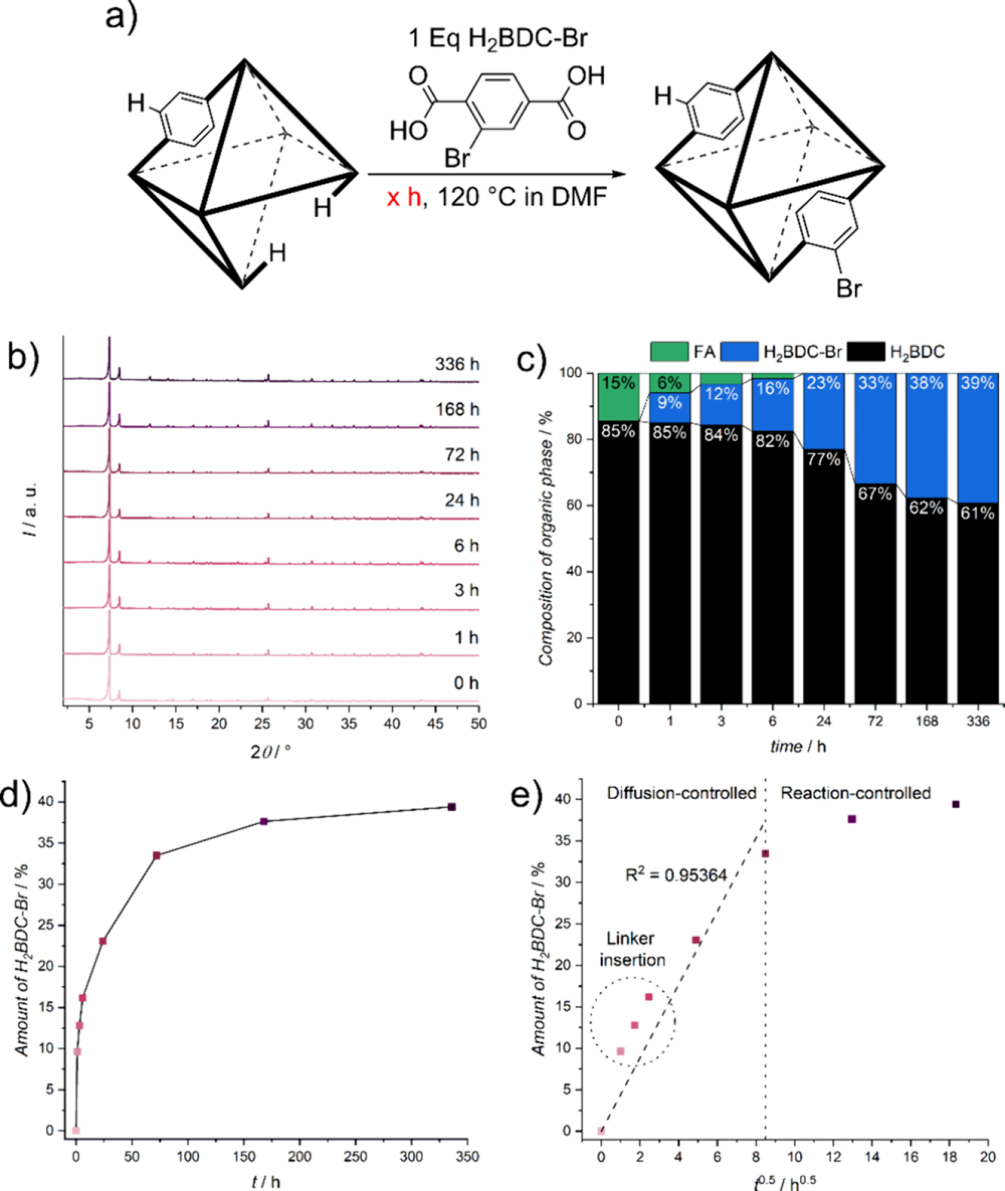
Kinetics of the PSE process over time illustrate the dynamic interplay between linker insertion and linker exchange, highlighting the maximum duration during which the PSE is primarily controlled by diffusion. (a) A schematic overview of the PSE reaction conditions is provided, with bonds ending with -H representing defects generated by incorporated FA. (b) PXRD patterns confirm the sustained high crystallinity of the nanoparticles after PSE. (c) Analysis of the composition of the organic part of the MOFs after PSE indicates the initial replacement of FA by the new linker followed by the subsequent exchange of the original linker. (d) The amount of incorporated linker versus the PSE time indicates rapid incorporation during the initial stages of the PSE, corresponding to the linker insertion into the defect sides. (e) A plot of the amount of incorporated linker against the square root of PSE time reveals a linear region during the first 72 h, indicative of the diffusion-controlled phase of the PSE. A deviation from this linear relationship occurs during the linker insertion phase, attributable to accelerated exchange into the defect sides. The colors used in (d) and (e) correspond to the exchange times presented in b).

To conduct a detailed kinetic investigation of the PSE process, we performed all PSE experiments on UiO-66 nanoparticles of a uniform particle size (approximately 350 nm), synthesized using 100 eq FA, to minimize the influence of the particle size on the diffusion-controlled PSE. The duration of PSE experiments ranged from 1 to 336 h to capture the complete progression of the process from its initial stages to full completion. Consistent with the setup described in the previous chapter, we maintained the other PSE conditions unchanged, as illustrated in the schematic representation in Figure 3a. Throughout the PSE time frame, the nanoparticles retained their high crystallinity, as evidenced by PXRD measurements (Figures 3b and S16), with no observable changes in particle size or morphology, as confirmed by SEM images (Figure S17).

We assessed the total composition of the organic part of the UiO-66 nanoparticles for the entire time frame using ^1^H NMR digestion experiments. The results of these experiments are detailed in Figure 3c, with additional data from ^1^H NMR measurements shown in Figure S18 and Table S3. Consistent with the literature, our findings suggest that the replacement of FA predominantly occurs during the early stages of the PSE,^8,12^ up to a PSE time of 6 h. Between 6 and 24 h, the linker insertion reaches completion and linker exchange becomes the dominant process. Subsequently, the postsynthetic linker exchange ensues, with the original linker (H_2_BDC) gradually being replaced by the new linker (H_2_BDC-Br), with substantial exchange occurring up to 72 h. From 72 to 168 h, only a marginal increase in linker exchange is observed, while after 336 h, minimal additional linker exchange occurs, indicating that the system approaches thermodynamic equilibrium with approximately 39% of the new linker incorporated.

Investigating the porosity using argon physisorption measurements (see Figure S19), we observed that the porosity remains intact even after exchange times up to 336 h, while the BET-surface area decreases linearly with the amount of newly incorporated linker (H_2_BDC-Br). Additionally, the pore size distribution shows a shift from 14 to 12 Å during the initial stages of the PSE, up to 24 h, which correlates well with the linker insertion step. This shift can be attributed to the new linker replacing FA in missing cluster defects, a phenomenon that is consistent with the literature, suggesting a 1:1 ratio of replaced FA to incorporate new linker during insertion into these defects (as seen in Figure 3c) for 0 and 1 h).^12^ From 24 to 336 h, the pore size distribution remains relatively stable, indicating minimal changes during the linker exchange phase.

The plot of the amount of incorporated linker versus time (refer to Figure 3d) demonstrates a rapid exchange rate in the early stages of the PSE. This can be attributed primarily to two factors: First, there is a decrease in the concentration gradient caused by a reduced free linker concentration in the solution as the PSE progresses. This is particularly significant given the absence of an excess of linker. Second, the rate of linker insertion is faster than that of linker exchange, a phenomenon supported by previous studies, despite these experiments being conducted on samples of varying particle sizes, which, as we have shown, can influence the outcome.^8,12^

To further explore the kinetics of the linker insertion and the diffusion-controlled process of the PSE, we plotted the amount of incorporated linker versus the square root of the time in Figure 3e). Up to 8 h^0.5^ or 72 h, the incorporation rate increases nearly linearly with the square root of the time, indicated by a strong *R*^2^ value of 0.95. This linear relationship suggests diffusion limitation, consistent with observations from previous studies on PSE in ZIFs.^10^ The values for the first 1 to 6 h slightly exceed the linear regression line, which we attribute to the faster rate of linker insertion into defects compared to linker exchange, as further elucidated in subsequent analysis.

It also becomes evident from the data that for reaction times longer than 72 or 8 h^0.5^, the PSE transitions from being diffusion-limited to more reaction-controlled. This leads us to conclude that under these PSE conditions, short PSE times up to 72 h are likely to result in the formation of core–shell structured UiO-66 nanoparticles, while longer times may lead to a more homogeneous distribution of the linker within the framework, in line with findings in previous studies.^11^ In general, these results lead us to the conclusion that for a true investigation of the diffusion limitation on the PSE process, the linker exchange must be investigated in a system in which no linker insertion is possible. This must be done while retaining the original particle size due to its influence on the PSE. To achieve this, we utilized PSE to incorporate the same type of linker (H_2_BDC) already present in the framework. This approach effectively inserted the linker into the defect sites, resulting in FA-free UiO-66 nanoparticles with an unchanged particle size.

The schematic of the synthesis conditions is outlined in Figure S20, with detailed synthesis conditions provided in the SI, Section 1. This method successfully achieved a complete exchange of FA, as confirmed by ^1^H NMR digestion experiments (Figure S21), while retaining high crystallinity, porosity, particle size, and morphology (Figures S22–S24).

**Figure 4:**
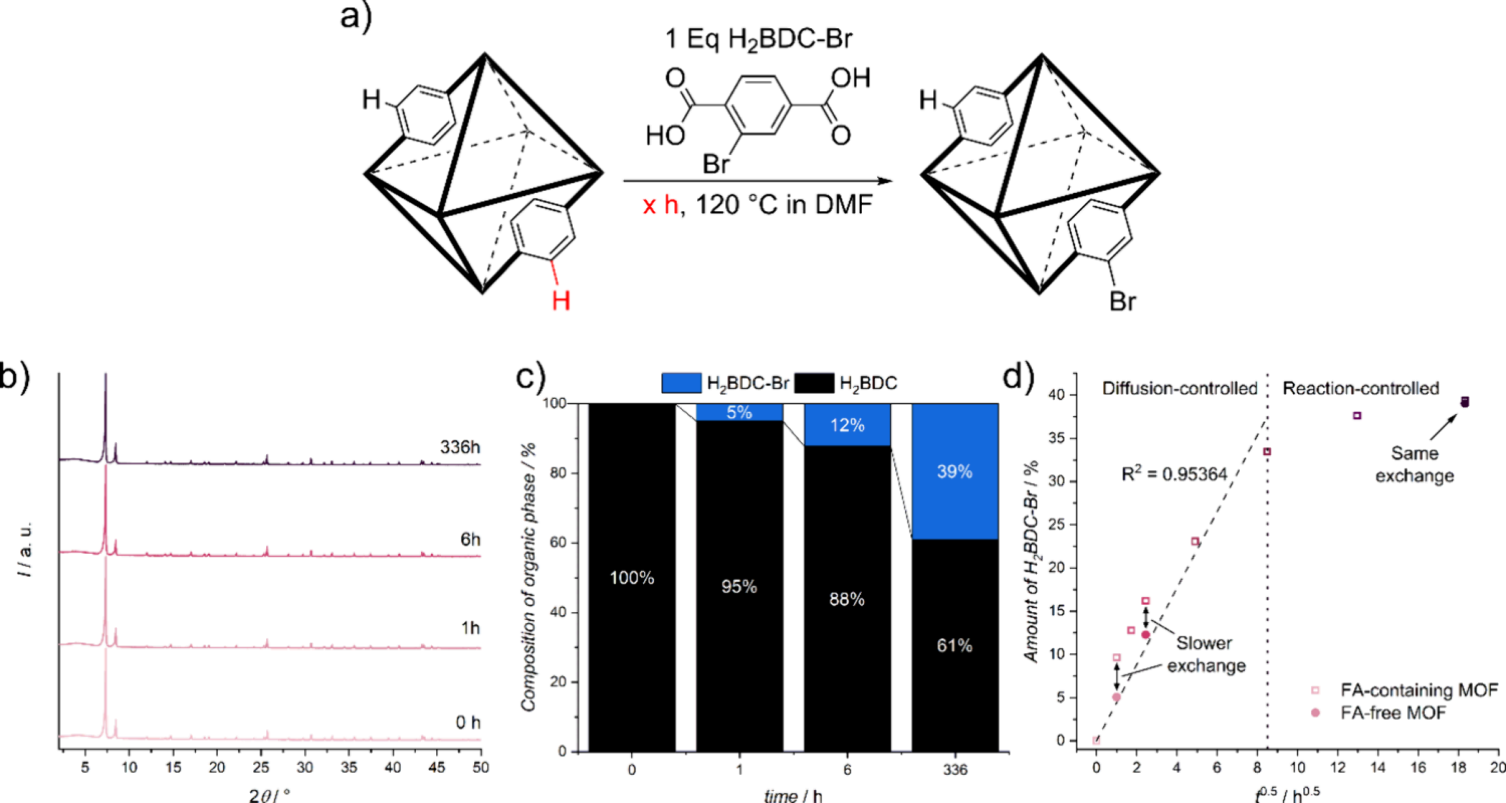
PSE on FA-free UiO-66 nanoparticles shows that the linker insertion of H_2_BDC-Br into the defects sides previously occupied by FA is faster than the linker exchange. (a) Schematic overview of the PSE procedure. (b) Comparison of PXRDs after the two PSE procedures indicating that the crystallinity is retained. (c) Comparison of the composition of the organic part measured via ^1^H NMR dissolution experiments. (d) Illustrates that the rate of linker insertion into defects is faster than the exchange of the linker by comparing the amount of incorporated linker against the square root of PSE time. The amount of incorporated linker for the FA-free MOF lies closer to the linear fit, indicating that the process is truly diffusion-controlled. The □ symbols represent the amount of incorporated H_2_BDC-Br for the FA-containing MOFs shown in Figure 3e, while the ● symbols represent the amount for FA-free MOFs shown in this figure.

With these FA-free UiO-66 nanoparticles, we proceeded with the same PSE using H_2_BDC-Br, aiming to gain a more detailed understanding of the kinetics of the exchange process. In particular, we focused on short PSE reaction times to determine if linker insertion occurs more rapidly than pure linker exchange. Accordingly, we performed the PSE for 1 h (1 h^0.5^) and 6 h (2.45 h^0.5^). Long PSE reaction times (336 or 18.3 h^0.5^) were also investigated to assess changes in the exchange rate as the system approaches thermodynamic equilibrium. The schematic of these PSE conditions is shown in Figure 4a.

We demonstrate that after the PSE with H_2_BDC-Br, crystallinity was maintained (Figures 4b and S25), and the new linker could be incorporated into the framework even without the presence of FA (Figure 4c). Furthermore, the particle size and morphology remained unchanged post-PSE, while the BET-surface area decreased linearly with increasing amounts of incorporated H_2_BDC-Br, as determined from ^1^H NMR digestion experiments (refer to Figures S26–S28 and Table S4). Notably, PSE with shorter reaction times exhibited a substantial reduction in the incorporation of the new linker (H_2_BDC-Br) into the FA-free UiO-66 compared to the FA-containing UiO-66 (Figure 4d). This variation cannot be attributed to differences in particle size, as the particles used in these experiments were uniformly sized.

The different exchange rates observed during the PSE can be explained by two main factors: First, the differing reactivity of linker insertion versus linker exchange, as supported by computational simulations;^15^ second, the enhanced diffusion through FA-containing UiO-66, which features larger pores compared to FA-free UiO-66 (Figure S24). This explanation aligns with the linear relationship observed in the exchange rates, highlighting that the pure linker exchange is largely governed by diffusion dynamics (see Figure 4d). Thus, our method effectively investigates the PSE process, focusing on diffusion-controlled conditions while minimizing the impact of particle size. Further, we demonstrate that the exchange rate near the thermodynamic equilibrium (336 or 18.3 h^0.5^) remains unchanged and the defects do not influence the total exchange but solely impact the kinetics of the linker exchange.

In essence, our findings prompt us to inquire about the specific location of the defects in the UiO-66 particle. We conducted a linker exchange process under diffusion-controlled PSE conditions, anticipated to lead to a core–shell structure, as elucidated in the subsequent section. Notably, our results show that FA is exchanged initially, aligning with prior observations. Further, Marti-Gastaldo and co-workers demonstrate for micrometer-sized Zr-MOF crystals, synthesized through the modulation approach, that a core–shell structure emerged for short PSE reaction times,^11^ although they did not investigate at which stage the modulator gets removed. We propose that the defects likely reside close to the particle surface. This hypothesis is supported by crystal growth principles. These principles suggest that the outermost layer of the particles, being the last to form during the synthesis, may not have undergone a complete exchange of FA from the linker.

Overall, our findings underscore our conclusion that the linker insertion into the defects occurs as the initial step in the process, emphasizing the strategic importance of the defects in influencing the PSE process during the early stages.

### Calculating the Minimum Incorporation Depth of UiO-66 Core–Shell Nanoparticles

**Figure 5:**
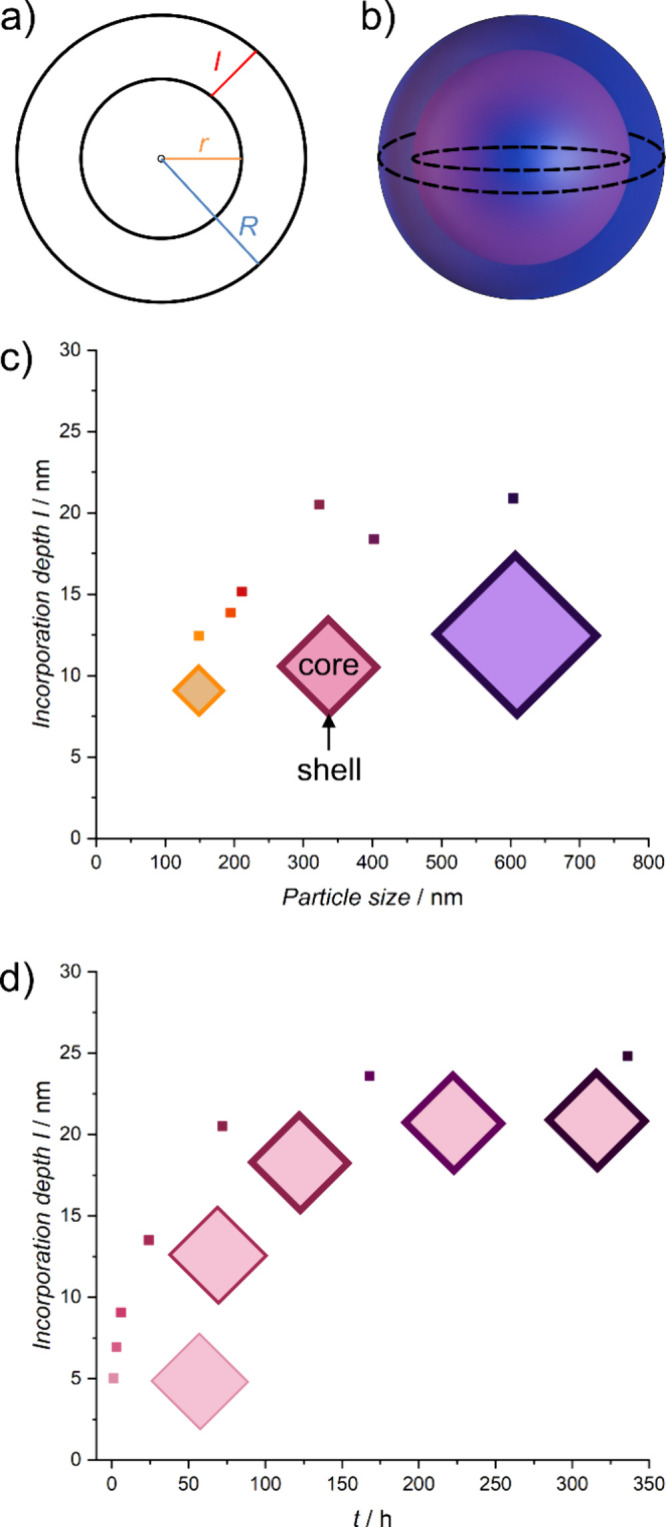
Calculation of the minimum incorporation depth using a spherical shell model for UiO-66 nanoparticles, varying in particle size and PSE reaction time. (a, b) Display 2D and 3D representations of the spherical shell model, respectively, featuring *r* as the core radius, *R* as the radius of the core–shell particle, and *I* as the minimum incorporation depth or minimum shell thickness. (c) Illustrates the plot of minimum incorporation depth against particle size and (d) shows it against the PSE reaction time. The schematic octahedra in each panel illustrate the difference in incorporation depth in relation to the particle size. The color used in (c) corresponds to the samples discussed in Figure 2, and the color in (d) corresponds to the samples discussed in Figure 3.

In the previous chapters, we demonstrated that our PSE conditions facilitate a diffusion-controlled exchange process, ideally resulting in core–shell UiO-66 nanoparticles. In this section, we describe a method for calculating the minimum incorporation depth of the new linker in the UiO-66 nanoparticles. Subsequently, we present a proof of principle measurement that directly showcases the linker distribution. To calculate the minimum incorporation depth (*I*_min,calc._) only two experimentally determinable properties of the MOF particles are required: particle size and total composition of the organic part. Particle size and morphology can be routinely determined using conventional imaging techniques such as SEM or TEM, with SEM images utilized in our study. The total composition of the organic part can be assessed through standard ^1^H NMR digestion experiments. We applied a calculation method assuming a complete exchange within a spherical shell (refer to Figure 5a,b). Detailed calculations are provided in SI in Section 4 and Figure S29. This method enables the calculation of the minimum incorporation depth, which could not be measured on nanoparticles due to the size limitation imposed by commonly used techniques.

Based on our study presented in the first chapter, Figure 5c illustrates that the minimum incorporation depth for UiO-66 nanoparticles varies from 10 to 20 nm across different sizes. The incorporation depth appears notably small relative to the overall particle size—specifically, it is only 20 nm for the largest particles, which measure 600 nm. This disproportion can be rationalized by the volume increase being proportional to *r*^3^, resulting in a substantial volume for the shell despite its seemingly low minimum incorporation depth. Generally, the minimum incorporation depth exhibits minimal change with increasing particle size, which is consistent with the diffusion-limitation of the PSE process. Larger particles tend to show a slight increase in incorporation depth, likely due to a higher concentration of defects on such particles.

Building upon the findings in the second chapter, Figure 5d captures the dynamic progression of the minimum incorporation depth over time during the PSE process. The depth increases rapidly from 5 nm after 1 h to 20 nm by 72 h and then plateaus, reaching 25 nm by 336 h. This trend aligns well with the diffusion and reaction-limited PSE process, which were detailed in the previous chapter. Using an octahedral shell as a model, the same trend is observed (see Figure S30). The minimum incorporation depth for all presented UiO-66 core–shell nanoparticles is summarized in Table S5.

While our model allows the calculation of the minimum incorporation depth, it does not account for the gradient of linker incorporation within the shell. To address this limitation, we present a proof-of-principle measurement specifically designed to directly measure the linker distribution in the UiO-66 core–shell nanoparticles.

**Figure 6:**
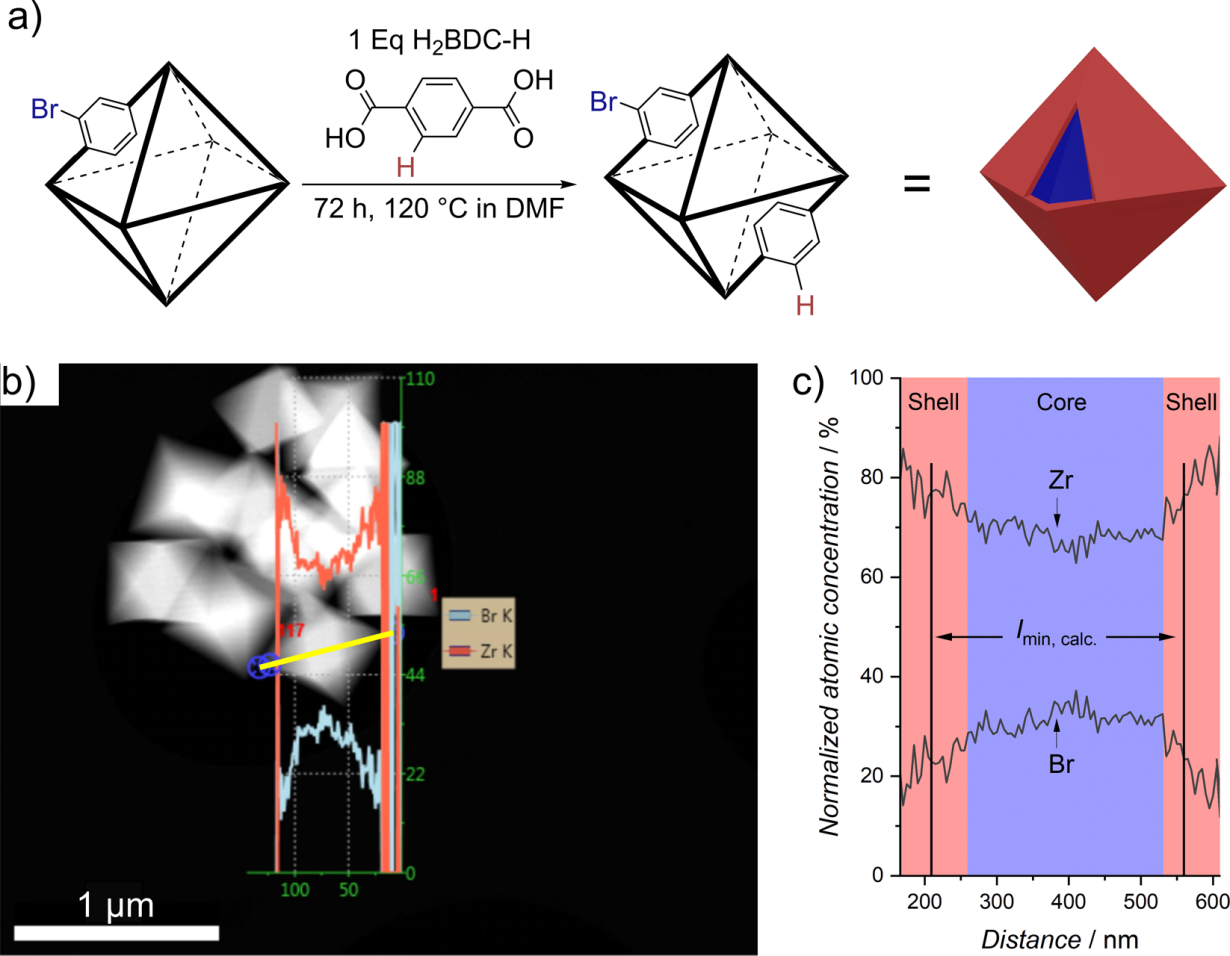
Direct measurement of the linker distribution in UiO-66 nanoparticles using STEM-EDX line scan showing a core–shell structure. (a) Schematic representation of the PSE conditions employed for synthesizing UiO-66 core–shell particles. (b) STEM-image displaying UiO-66 nanoparticles after PSE with the highlighted EDX line scan in yellow alongside the normalized atomic concentration of Zr and Br. (c) Graph illustrating the normalized atomic concentration of Zr to Br along the STEM-EDX line scan, emphasizing the core–shell structure of the nanoparticles, alongside the calculated minimum incorporation depth *I*_min,calc._ derived from the spherical shell model.

We employed transmission electron microscopy combined with energy-dispersive X-ray spectroscopy (TEM-EDX) to directly assess the linker distribution and incorporation depth of the new linker introduced by PSE. To facilitate this measurement, we synthesized UiO-66 core–shell nanoparticles with the bromine linker (H_2_BDC-Br) in the core, as illustrated in Figure 6a. This synthesis was performed under the same PSE conditions previously used, but with UiO-66-Br as the initial MOF and introducing H_2_BDC-H with the PSE. This choice of a core–shell arrangement for the linker serves two purposes. First, when the original MOF contains the bromine linker, the bromine content in the core–shell MOF is higher, given the maximum exchange rate under these PSE conditions is 50%. Second, the localization of bromine primarily in the core simplifies the measurement of its distribution using STEM-EDX compared to bromine, which is distributed in the shell and therefore measured throughout the entire particle.

The characterization of the UiO-66-Br nanoparticles before and after PSE is detailed in Figures S31–S36 and Table S6. Throughout the process, the crystallinity, particle size, and morphology were maintained, while porosity increased. Remarkably, approximately 40% of the core–shell MOF was constituted by the new linker (H_2_BDC). Despite the relatively large particle size of approximately 500 nm, the exchange rate is comparably high, attributed to the functional groups and their influence on the exchange rate, as demonstrated in previous studies.^14^

Subsequently, we performed a STEM-EDX line scan from corner to corner of the UiO-66 core–shell nanoparticle, measuring the atomic concentration throughout the particle (Figure 6b). The raw data from this line scan, including all measured elements, is provided in Figure S37. For clarity in visualizing the linker distribution, we focused only on the concentration of bromine and zirconium. We assumed a uniform distribution of Zr throughout the particle using it as a baseline for comparison—a method consistent with XPS investigations.^11,17^ We normalized the combined atomic concentration of Zr and Br to 100% and plotted the individual atomic concentration throughout the line scan in Figure 6c. Ideally, if the bromine linker were homogeneously distributed, the atomic concentrations of Zr and Br would remain constant across the line scan. However, our observations deviate from this ideal: the intensity of the Br signal drastically decreases at the shell of the particle, which directly indicates the presence of a core–shell structure of the UiO-66 nanoparticles.

We also applied our presented method to calculate the minimum incorporation depth for these UiO-66 core–shell nanoparticles (refer to Table S6), resulting in a minimum incorporation depth of roughly 40 nm (shown as black lines in Figure 6c). This value contrasts with the actual incorporation depth estimated from the STEM-EDX line scan, which ranges between 75 and 100 nm. This discrepancy arises because our calculations assumed complete exchange within the shell, a condition that is rarely fully achieved in practice. As already shown for micrometer-sized Zr-MOF crystals, a gradient within the shell and therefore a thicker shell than calculated will form.^13,16^ This phenomenon is similarly observed in UiO-66 nanoparticles. Hence, while useful, the minimum incorporation depth we calculate should be regarded as a conservative estimate.

In summary, we present the first direct measurement of linker distribution in MOF nanoparticles after PSE. Our results confirm the emergence that a core–shell structure, previously observed only in micrometer-sized crystals, also occurs in nanoparticles under diffusion-controlled PSE conditions. Additionally, we introduce a method for estimating the minimum incorporation depth within these core–shell nanoparticles.

## Conclusions

Our study demonstrates the pronounced influence of particle size on the linker exchange in PSE on UiO-66 nanoparticles. By utilizing diffusion-limited reaction conditions, we found that larger nanoparticles exhibit significantly lower exchange rates, attributable to their higher volume-to-surface ratio. Additionally, we examine the influence of defects on PSE by comparing exchange rates in nanoparticles of identical particle size with and without FA in the structure. Through PSE using the intrinsic MOF linker, we successfully substituted FA from the UiO-66 nanoparticles without altering their size. Furthermore, by conducting PSE on particles both with and without FA in the framework, we demonstrate that linker integration into the framework precedes linker exchange. Our results also confirm that the linker exchange in FA-free UiO-66 nanoparticles is truly diffusion-controlled.

Additionally, we developed a geometric model that enables the calculation of the minimum incorporation depth of core–shell MOFs. For this model, only the particle size, morphology, and composition of the organic part are needed—information readily available from routine measurements such as SEM and ^1^H NMR dissolution experiments. We observed relatively consistent minimum incorporation rates for UiO-66 nanoparticles of increasing size, and we illustrate the growth of the minimum incorporation depth with prolonged PSE reaction times. This approach is considered to be transferable to other MOF core–shell systems where the composition of the organic part and particle size are quantifiable. However, it is important to note that UiO-66 is a comparatively microporous MOF, which may encounter diffusion limitations, especially when exchanging linkers that include larger functional groups, such as bromine, as demonstrated in our experiments. While our results should be highly applicable to UiO-66 and its derivatives studied, the dynamics might differ in MOFs, where larger pore windows could minimize diffusion barriers. Despite these variations, given the widespread use of UiO-66 and its numerous derivatives, our insights are likely to find practical applications in a variety of settings.

Furthermore, through a proof-of-principle experiment utilizing STEM-EDX measurements, we directly measure the linker distribution and actual incorporation depth within the nanoparticles. While our calculation method estimated the minimum incorporation depth at 40 nm, it slightly underestimated the actual depth, which ranged from 75 to 100 nm. The discrepancy is attributed to a gradient in the linker incorporation within the shell.

Collectively, our findings enhance the understanding of the PSE for MOF nanoparticles and introduce a straightforward method for calculating the minimum shell thickness in core–shell MOFs. This holds significant promise for future investigations into core–shell MOFs and their potential applications.

## Supporting Information Available

Additional experimental details; materials; detailed description for the calculation of the minimum incorporation depth; detailed characterization of the MOFs, particle size distributions, DLS spectra, argon-sorption isotherms and pore size distributions, ^1^H NMR spectra, SEM images, PXRD spectra, and STEM-EDX raw data (PDF)

## References

[ref1] Freund R.; Canossa S.; Cohen S. M.; Yan W.; Deng H.; Guillerm V.; Eddaoudi M.; Madden D. G.; Fairen-Jimenez D.; Lyu H.; Macreadie L. K.; Ji Z.; Zhang Y.; Wang B.; Haase F.; Wöll C.; Zaremba O.; Andreo J.; Wuttke S.; Diercks C. S. 25 Years of Reticular Chemistry. Angew. Chem., Int. Ed. 2021, 60, 23946–23974. 10.1002/anie.202101644. 33783111

[ref2] Cohen S. M. The Postsynthetic Renaissance in Porous Solids. J. Am. Chem. Soc. 2017, 139, 2855–2863. 10.1021/jacs.6b11259. 28118009

[ref3] Karagiaridi O.; Bury W.; Mondloch J. E.; Hupp J. T.; Farha O. K. Solvent-assisted linker exchange: an alternative to the de novo synthesis of unattainable metal-organic frameworks. Angew. Chem., Int. Ed. 2014, 53, 4530–4540. 10.1002/anie.201306923. 24652755

[ref4] Fluch U.; Paneta V.; Primetzhofer D.; Ott S. Uniform distribution of post-synthetic linker exchange in metal-organic frameworks revealed by Rutherford backscattering spectrometry. Chem. Commun. 2017, 53, 6516–6519. 10.1039/C7CC02631E. PMC5846729 28573305

[ref5] Woo H.; Devlin A. M.; Matzger A. J. In Situ Observation of Solvent Exchange Kinetics in a MOF with Coordinatively Unsaturated Sites. J. Am. Chem. Soc. 2023, 145, 18634–18641. 10.1021/jacs.3c06396. 37552873

[ref6] Du Bois D. R.; Matzger A. J. Reagent Reactivity and Solvent Choice Determine Metal-Organic Framework Microstructure during Postsynthetic Modification. J. Am. Chem. Soc. 2021, 143, 671–674. 10.1021/jacs.0c12040. 33382943

[ref7] Dodson R. A.; Kalenak A. P.; Matzger A. J. Solvent Choice in Metal-Organic Framework Linker Exchange Permits Microstructural Control. J. Am. Chem. Soc. 2020, 142, 20806–20813. 10.1021/jacs.0c10224. 33237750

[ref8] Marreiros J.; Caratelli C.; Hajek J.; Krajnc A.; Fleury G.; Bueken B.; de Vos D. E.; Mali G.; Roeffaers M. B. J.; van Speybroeck V.; Ameloot R. Active Role of Methanol in Post-Synthetic Linker Exchange in the Metal–Organic Framework UiO-66. Chem. Mater. 2019, 31, 1359–1369. 10.1021/acs.chemmater.8b04734.

[ref9] Boissonnault J. A.; Wong-Foy A. G.; Matzger A. J. Core-Shell Structures Arise Naturally During Ligand Exchange in Metal-Organic Frameworks. J. Am. Chem. Soc. 2017, 139, 14841–14844. 10.1021/jacs.7b08349. 29020774

[ref10] Jayachandrababu K. C.; Sholl D. S.; Nair S. Structural and Mechanistic Differences in Mixed-Linker Zeolitic Imidazolate Framework Synthesis by Solvent Assisted Linker Exchange and de Novo Routes. J. Am. Chem. Soc. 2017, 139, 5906–5915. 10.1021/jacs.7b01660. 28388071

[ref11] Lerma-Berlanga B.; Ganivet C. R.; Almora-Barrios N.; Tatay S.; Peng Y.; Albero J.; Fabelo O.; González-Platas J.; García H.; Padial N. M.; Martí-Gastaldo C. Effect of Linker Distribution in the Photocatalytic Activity of Multivariate Mesoporous Crystals. J. Am. Chem. Soc. 2021, 143, 1798–1806. 10.1021/jacs.0c09015. 33432818

[ref12] Taddei M.; Wakeham R. J.; Koutsianos A.; Andreoli E.; Barron A. R. Post-Synthetic Ligand Exchange in Zirconium-Based Metal-Organic Frameworks: Beware of The Defects!. Angew. Chem., Int. Ed. 2018, 57, 11706–11710. 10.1002/anie.201806910. 29989290

[ref13] Al Danaf N.; Schrimpf W.; Hirschle P.; Lamb D. C.; Ji Z.; Wuttke S. Linker Exchange via Migration along the Backbone in Metal-Organic Frameworks. J. Am. Chem. Soc. 2021, 143, 10541–10546. 10.1021/jacs.1c04804. 34228932

[ref14] Park H.; Kim S.; Jung B.; Park M. H.; Kim Y.; Kim M. Defect Engineering into Metal-Organic Frameworks for the Rapid and Sequential Installation of Functionalities. Inorg. Chem. 2018, 57, 1040–1047. 10.1021/acs.inorgchem.7b02391. 29303561

[ref15] Chiu C.-C.; Shieh F.-K.; Tsai H.-H. G. Ligand Exchange in the Synthesis of Metal-Organic Frameworks Occurs Through Acid-Catalyzed Associative Substitution. Inorg. Chem. 2019, 58, 14457–14466. 10.1021/acs.inorgchem.9b01947. 31498604

[ref16] McCarthy B. D.; Liseev T.; Sortica M. A.; Paneta V.; Gschwind W.; Nagy G.; Ott S.; Primetzhofer D. Elemental Depth Profiling of Intact Metal-Organic Framework Single Crystals by Scanning Nuclear Microprobe. J. Am. Chem. Soc. 2021, 143, 18626–18634. 10.1021/jacs.1c08550. 34726402 PMC8587607

[ref17] Moreton J. C.; Low J. X.; Penticoff K. C.; Cohen S. M.; Benz L. An X-ray Photoelectron Spectroscopy Study of Postsynthetic Exchange in UiO-66. Langmuir 2022, 38, 1589–1599. 10.1021/acs.langmuir.1c03015. 35029998

[ref18] Tu M.; Wannapaiboon S.; Fischer R. A. Inter-conversion between zeolitic imidazolate frameworks: a dissolution–recrystallization process. J. Mater. Chem. A 2020, 8, 13710–13717. 10.1039/D0TA02975K.

[ref19] Abraha Y. W.; Jacobs F. J. F.; Brink A.; Langner E. H. G. Effect of Solvent Assisted Linker Exchange (SALE) and De Novo Synthetic Routes on CO2 Uptake and Fixation by Mixed-Linker Zeolitic Imidazolate Frameworks. J. Inorg. Organomet. Polym. Mater. 2023, 33, 2058–2074. 10.1007/s10904-023-02653-5.

[ref20] Krajnc A.; Kos T.; Zabukovec Logar N.; Mali G. A Simple NMR-Based Method for Studying the Spatial Distribution of Linkers within Mixed-Linker Metal-Organic Frameworks. Angew. Chem., Int. Ed. 2015, 54, 10535–10538. 10.1002/anie.201504426. 26178577

[ref21] Shi Le; Kobylarczyk J.; Kwiatkowski K.; Sieklucka B.; Ferlay S.; Podgajny R. Binary and Ternary Core–Shell Crystals of Polynuclear Coordination Clusters via Epitaxial Growth. Cryst. Growth Des. 2022, 22, 3413–3420. 10.1021/acs.cgd.2c00216.

[ref22] Liu C.; Bao T.; Yuan L.; Zhang C.; Wang J.; Wan J.; Yu C. Semiconducting MOF@ZnS Heterostructures for Photocatalytic Hydrogen Peroxide Production: Heterojunction Coverage Matters. Adv. Funct. Mater. 2022, 32, 2111404 10.1002/adfm.202111404.

[ref23] He Y.; Sun M.; Zhao Q.; Shang J.; Tian Y.; Xiao P.; Gu Q.; Li L.; Webley P. A. Effective Gas Separation Performance Enhancement Obtained by Constructing Polymorphous Core-Shell Metal-Organic Frameworks. ACS Appl. Mater. Interfaces 2019, 11, 30234–30239. 10.1021/acsami.9b08592. 31339300

[ref24] Abraha Y. W.; Tsai C.-W.; Niemantsverdriet J. W. H.; Langner E. H. G. Optimized CO2 Capture of the Zeolitic Imidazolate Framework ZIF-8 Modified by Solvent-Assisted Ligand Exchange. ACS Omega 2021, 6, 21850–21860. 10.1021/acsomega.1c01130. 34497880 PMC8412924

[ref25] Fu G.; Wu P.; Zhang S.; Wang L.; Xu M.; Huai X. Improvement of water adsorption performance of UiO-66 by post-synthetic modification. Dalton Trans. 2023, 52, 11671–11678. 10.1039/D3DT01062G. 37552108

[ref26] Kim S.; Lee J.; Jeoung S.; Moon H. R.; Kim M. Surface-Deactivated Core-Shell Metal-Organic Framework by Simple Ligand Exchange for Enhanced Size Discrimination in Aerobic Oxidation of Alcohols. Chem. - Eur. J. 2020, 26, 7568–7572. 10.1002/chem.202000933. 32096306

[ref27] Chu J.; Ke F.-S.; Wang Y.; Feng X.; Chen W.; Ai X.; Yang H.; Cao Y. Facile and reversible digestion and regeneration of zirconium-based metal-organic frameworks. Commun. Chem. 2020, 3, 5 10.1038/s42004-019-0248-7. 36703351 PMC9812265

[ref28] Schaate A.; Roy P.; Godt A.; Lippke J.; Waltz F.; Wiebcke M.; Behrens P. Modulated synthesis of Zr-based metal-organic frameworks: from nano to single crystals. Chem. - Eur. J. 2011, 17, 6643–6651. 10.1002/chem.201003211. 21547962

[ref29] Shan B.; McIntyre S. M.; Armstrong M. R.; Shen Y.; Mu B. Investigation of Missing-Cluster Defects in UiO-66 and Ferrocene Deposition into Defect-Induced Cavities. Ind. Eng. Chem. Res. 2018, 57, 14233–14241. 10.1021/acs.iecr.8b03516.

