# Supplementary material for: A Method for Determining Incorporation Depth in Core–Shell UiO-66 Nanoparticles Synthesized Via Postsynthetic Exchange
Source: Inorg Chem. 2024 Jun 12;63(25):11897–906. doi: 10.1021/acs.inorgchem.4c01787 (PMC11200254; doi:10.1021/acs.inorgchem.4c01787)
Supplement: Supplementary file 1 — ic4c01787_si_001.pdf [file ic4c01787_si_001.pdf]

Supporting Information

A Method for Determining Incorporation Depth in Core-Shell UiO-66 Nanoparticles Synthesized via Postsynthetic Exchange

Adrian Hannebauer,[a] Yaşar Krysiak,[a,b] Andreas Schaate[a,c,*]

[a] Institute of Inorganic Chemistry, Leibniz University Hannover, Callinstraße 9, 30167 Hannover, Germany

[b] Laboratory of Nano and Quantum Engineering, Leibniz University Hannover, Schneiderberg 39, 30167 Hannover, Germany

[c] Cluster of Excellence PhoenixD (Photonics, Optics and Engineering – Innovation Across Disciplines), Leibniz University Hannover, Welfengarten 1A, 30167, Hannover, Germany

*Corresponding author: Email: andreas.schaate@acb.uni-hannover.de

Table of Contents

Section 1 Experimental Details S3

Materials S3

Synthesis of UiO-66 and UiO-66-Br nanoparticles S3

Postsynthetic ligand exchange on UiO-66 and UiO-66-Br nanoparticles S5

Section 2 Particle size influence on the exchange rate of PSE S8

Characterization of UiO-66 nanoparticles before the PSE S8

Characterization of UiO-66 nanoparticles after the PSE S12

Section 3 Kinetic investigation of linker insertion versus linker exchange S22

PSE on FA-containing UiO-66 nanoparticles S22

PSE with H_2BDC-H to synthesize FA-free UiO-66 nanoparticles with H_2BDC-H S25

PSE on UiO-66 nanoparticles without formic acid S28

Section 4 Calculating the minimum incorporation depth of UiO-66 core-shell nanoparticles S32

Calculation of the minimum incorporation depth S32

Characterization of UiO-66-Br_H core-shell nanoparticles S34

STEM-EDX measurement on UiO-66-Br_H core shell nanoparticles S39

Section 1 Experimental Details

Materials

Zirconium (IV) chloride (>99.5%, $ZrCl_4$, Sigma Aldrich), *N,N*-dimethylformamide (99.8%, DMF, Sigma Aldrich), formic acid (>98%, HCOOH, Merck), terephthalic acid (99%, $C_8H_6O_4$ (H_2BDC), Honeywell), 2-bromoterephthalic acid (97%, $C_8H_5BrO_4$ (H_2BDC-Br), ABCR), ethanol (96%, C_2H_5OH, Merck), ammonium carbonate (Reag. Ph Eur, $(NH_4)_2CO_3$, Merck) deuterated water for NMR analysis (99,98% D, D_2O, Sigma Aldrich), were used without purification.

Synthesis of UiO-66 and UiO-66-Br nanoparticles

Synthesis of UiO-66 nanoparticles with increasing particle size

UiO-66_10FA

UiO-66_10FA was synthesized in 100 mL Pyrex glass vessels by sequentially dissolving 0.1505 g $ZrCl_4$ (0.646 mmol, 1eq), 0.244 mL formic acid (6.46 mmol, 10 eq), 0.047 mL demineralized water (2.58 mmol, 4 eq) and 0.1073 g H_2BDC (0.646 mmol, 1eq) in 25 mL DMF (322.89 mmol, 500 eq). The glass vessels were sealed and heated at 120 °C for 24 hours. After cooling to room temperature, the precipitate was separated by centrifugation, washed once with DMF and twice with ethanol centrifuging after each washing step to remove the supernatant. The precipitate was then dried under vacuum overnight. Finally, the obtained powders were purified by Soxhlet extraction with ethanol for 24 h and dried under vacuum.

UiO-66_25FA

UiO-66_25FA was synthesized in 100 mL Pyrex glass vessels by sequentially dissolving 0.1505 g $ZrCl_4$ (0.646 mmol, 1eq), 0.609 mL formic acid (16.14 mmol, 25 eq), 0.047 mL demineralized water (2.58 mmol, 4 eq) and 0.1073 g H_2BDC (0.646 mmol, 1eq) in 25 mL DMF (322.89 mmol, 500 eq). The glass vessels were sealed and heated at 120 °C for 24 hours. After cooling to room temperature, the precipitate was separated by centrifugation, washed once with DMF and twice with ethanol, centrifuging after each washing step to remove the supernatant. The precipitate was then dried under vacuum overnight. Finally, the obtained powders were purified by Soxhlet extraction with ethanol for 24 h and dried under vacuum.

UiO-66_50FA

UiO-66_50FA was synthesized in 100 mL Pyrex glass vessels by sequentially dissolving 0.1505 g $ZrCl_4$ (0.646 mmol, 1eq), 1.218 mL formic acid (32.29 mmol, 50 eq), 0.047 mL demineralized water (2.58 mmol, 4 eq) and 0.1073 g H_2BDC (0.646 mmol, 1eq) in 25 mL DMF (322.89 mmol, 500 eq). The glass vessels were sealed and heated at 120 °C for 24 hours. After cooling to room temperature, the precipitate was separated by centrifugation, washed once with DMF and twice with ethanol centrifuging after each washing step to remove the supernatant. The precipitate was then dried under vacuum overnight. Finally, the obtained powders were purified by Soxhlet extraction with ethanol for 24 h and dried under vacuum.

UiO-66_100FA

UiO-66_100FA was synthesized in 100 mL Pyrex glass vessels by sequentially dissolving 0.1505 g $ZrCl_4$ (0.646 mmol, 1eq), 2.44 mL formic acid (64.58 mmol, 10 eq), 0.047 mL demineralized water (2.58 mmol, 4 eq) and 0.1073 g H_2BDC (0.646 mmol, 1eq) in 25 mL DMF (322.89 mmol, 500 eq). The glass vessels were sealed and heated at 120 °C for 24 hours. After cooling to room temperature, the precipitate was separated by centrifugation, washed once with DMF and twice with ethanol, centrifuging after each washing step to remove the supernatant. The precipitate was then dried under vacuum overnight. Finally, the obtained powders were purified by Soxhlet extraction with ethanol for 24 h and dried under vacuum.

UiO-66_200FA

UiO-66_200FA was synthesized in 100 mL Pyrex glass vessels by sequentially dissolving 0.1505 g $ZrCl_4$ (0.646 mmol, 1eq), 4.87 mL formic acid (129.16 mmol, 200 eq), 0.047 mL demineralized water (2.58 mmol, 4 eq) and 0.1073 g H_2BDC (0.646 mmol, 1eq) in 25 mL DMF (322.89 mmol, 500 eq). The glass vessels were sealed and heated at 120 °C for 24 hours. After cooling to room temperature, the precipitate was separated by centrifugation, washed once with DMF and twice with ethanol, centrifuging after each washing step to remove the supernatant. The precipitate was then dried under vacuum overnight. Finally, the obtained powders were purified by Soxhlet extraction with ethanol for 24 h and dried under vacuum.

UiO-66_300FA

UiO-66_300FA was synthesized in 100 mL Pyrex glass vessels by sequentially dissolving 0.1505 g $ZrCl_4$ (0.646 mmol, 1eq), 7.309 mL formic acid (193.73 mmol, 300 eq), 0.047 mL demineralized

water (2.58 mmol, 4 eq) and 0.1073 g H_2BDC (0.646 mmol, 1eq) in 25 mL DMF (322.89 mmol, 500 eq). The glass vessels were sealed and heated at 120 °C for 24 hours. After cooling to room temperature, the precipitate was separated by centrifugation, washed once with DMF and twice with ethanol centrifuging after each washing step to remove the supernatant. The precipitate was then dried under vacuum overnight. Finally, the obtained powders were purified by Soxhlet extraction with ethanol for 24 h and dried under vacuum.

Synthesis of UiO-66-Br nanoparticles

UiO-66-Br nanoparticles were synthesized in 100 mL Pyrex glass vessels by sequentially dissolving 0.1505 g $ZrCl_4$ (0.646 mmol, 1 eq), 2.436 mL formic acid (64.58 mmol, 100 eq) and 0.1582 g H_2BDC-Br (0.646 mmol, 1 eq) in 25 mL DMF (322.9 mmol, 500 eq). The glass vessels were sealed and heated at 120 °C for 24 hours. After cooling to room temperature, the precipitate was separated by centrifugation, washed once with DMF and twice with ethanol centrifuging after each washing step to remove the supernatant. The precipitate was then dried under vacuum overnight. Finally, the obtained powders were purified by Soxhlet extraction with ethanol for 24 h and dried under vacuum.

Postsynthetic ligand exchange on UiO-66 and UiO-66-Br nanoparticles

PSE on UiO-66 nanoparticles with increasing particle size (UiO-66-H_Br)

The postsynthetic ligand exchange was performed in 100 mL Pyrex glass vessels. For this 0.063 g H_2BDC-Br (0.258 mmol, 1 eq) was dissolved in 10 mL DMF (129 mmol, 500 Eq) at room temperature. After dissolution, 0.0716 g UiO-66 (0.258 mmol of BDC, 1 Eq) was added to the solution and the vessels were sealed and heated at 120 °C for 72 h. After cooling to room temperature, the precipitate was separated by centrifugation, washed once with DMF and twice with ethanol centrifuging after each washing step to remove the supernatant. The precipitate was then dried under vacuum overnight. Finally, the obtained powders were purified by Soxhlet extraction with ethanol for 24 h and dried under vacuum.

For these syntheses the samples UiO-66_10FA, UiO-66_20FA, UiO-66_50FA, UiO-66_100FA, UiO-66_200FA and UiO-66_300FA were used.

PSE on UiO-66 nanoparticles with increasing time of PSE (UiO-66-H_Br)

The postsynthetic ligand exchange were performed in 100 mL Pyrex glass vessels. For this 0.063 g H_2BDC-Br (0.258 mmol, 1 eq) was dissolved in 10 mL DMF (129 mmol, 500 Eq) at room temperature. After dissolution, 0.0716 g UiO-66 (0.258 mmol of BDC, 1 Eq) was added to the solution and the vessels were sealed and heated at 120 °C for 1 – 336 h. For these syntheses, the UiO-66 nanoparticles synthesized with 100 Eq of formic acid were used. After cooling to room temperature, the precipitate was separated by centrifugation, washed once with DMF and twice with ethanol centrifuging after each washing step to remove the supernatant. The precipitate was then dried under vacuum overnight. Finally, the obtained powders were purified by Soxhlet extraction with ethanol for 24 h and dried under vacuum.

In order to minimize the effects of particle size on the PSE, only the sample UiO-66_100FA was used for these syntheses.

PSE on UiO-66-Br nanoparticles (UiO-66-Br_H)

The postsynthetic ligand exchange was performed in 100 mL Pyrex glass vessels. For this 0.0429 g H_2BDC (0.258 mmol, 1 eq) was dissolved in 10 mL DMF (129 mmol, 500 Eq) at room temperature. After dissolution 0.092 g UiO-66-Br (0.258 mmol of BDC-Br, 1 Eq) was added to the solution and the vessels were sealed and heated at 120 °C for 72 h. After cooling to room temperature, the precipitate was separated by centrifugation, washed once with DMF and twice with ethanol centrifuging after each washing step to remove the supernatant. The precipitate was then dried under vacuum overnight. Finally, the obtained powders were purified by Soxhlet extraction with ethanol for 24 h and dried under vacuum.

PSE on UiO-66 nanoparticles with H_2BDC (UiO-66-H_H)

The postsynthetic ligand exchange was performed in 100 mL Pyrex glass vessels. For this 0.043 g H_2BDC (0.258 mmol, 1 eq) was dissolved in 10 mL DMF (129 mmol, 500 Eq) at room temperature. After dissolution, 0.0716 g UiO-66 (0.258 mmol of BDC, 1 Eq) was added to the solution and the vessels were sealed and heated at 120 °C for 72 h. After cooling to room temperature, the precipitate was separated by centrifugation, washed once with DMF and twice with ethanol centrifuging after each washing step to remove the supernatant. The precipitate was then dried under vacuum overnight. Finally, the obtained powders were purified by Soxhlet extraction with ethanol for 24 h and dried under vacuum. For this synthesis, the UiO-66_100FA was used.

PSE on modified UiO-66 nanoparticles (UiO-66-H_H) with H_2BDC-Br (UiO-66-H_H_Br)

The postsynthetic ligand exchange was performed in 100 mL Pyrex glass vessels. For this 0.043 g H_2BDC (0.258 mmol, 1 eq) was dissolved in 10 mL DMF (129 mmol, 500 Eq) at room temperature. After dissolution, 0.0716 g UiO-66 (0.258 mmol of BDC, 1 Eq) was added to the solution and the vessels were sealed and heated at 120 °C for 1 h to 2 weeks. After cooling to room temperature, the precipitate was separated by centrifugation, washed once with DMF and twice with ethanol, centrifuging after each washing step to remove the supernatant. The precipitate was then dried under vacuum overnight. Finally, the obtained powders were purified by Soxhlet extraction with ethanol for 24 h and dried under vacuum.

For these syntheses, the modified UiO-66 nanoparticles (UiO-66-H_H) were used.

Section 2 Particle size influence on the exchange rate of PSE

Characterization of UiO-66 nanoparticles before the PSE

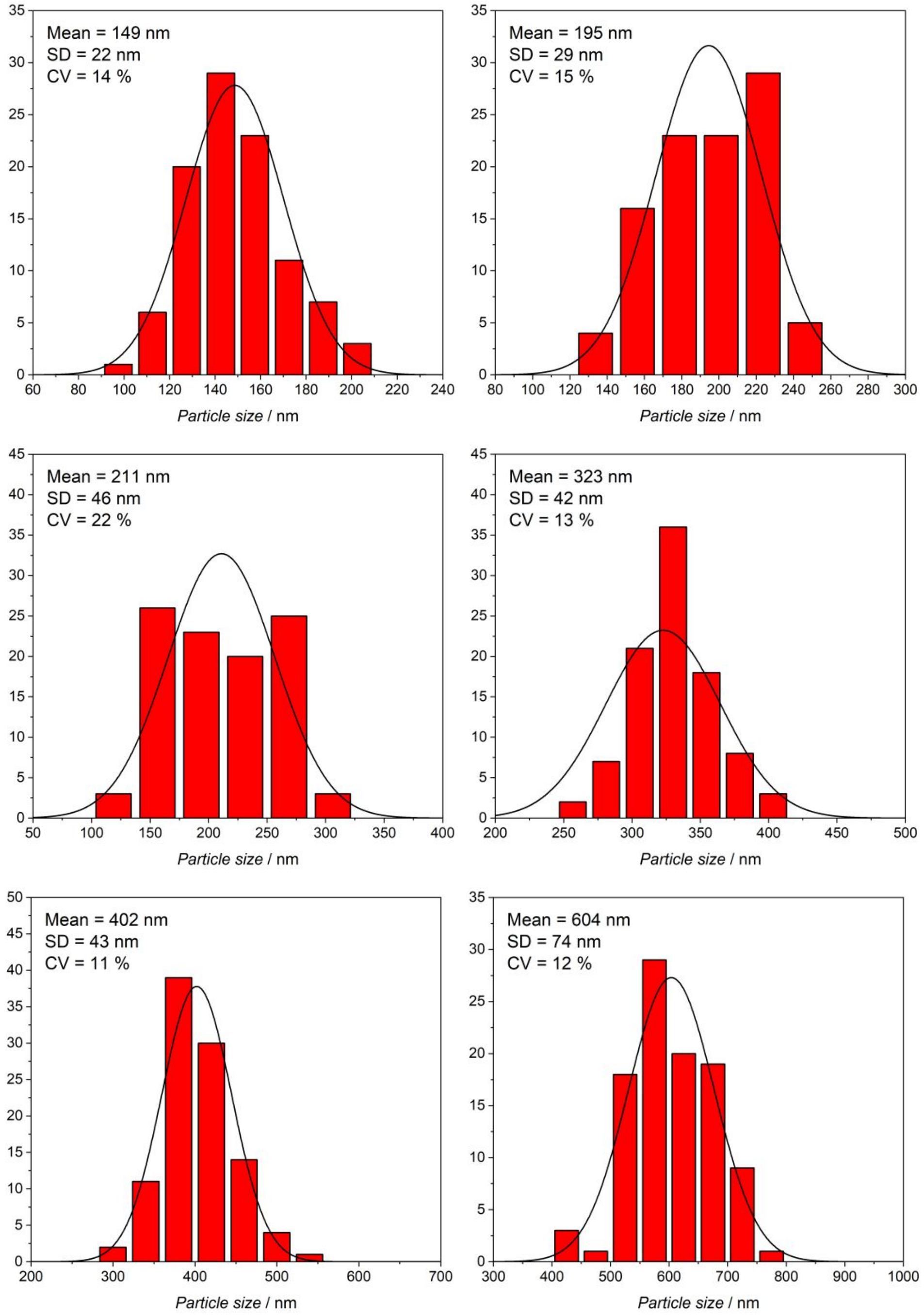

Figure S1. Particle size distribution from SEM images (100 particles measured each) of UiO-66 nanoparticles before the PSE. Top left: 10 Eq FA, top right: 25 Eq FA, middle left: 50 Eq FA, middle right: 100 Eq FA, bottom left: 200 Eq FA, bottom right: 300 Eq FA.

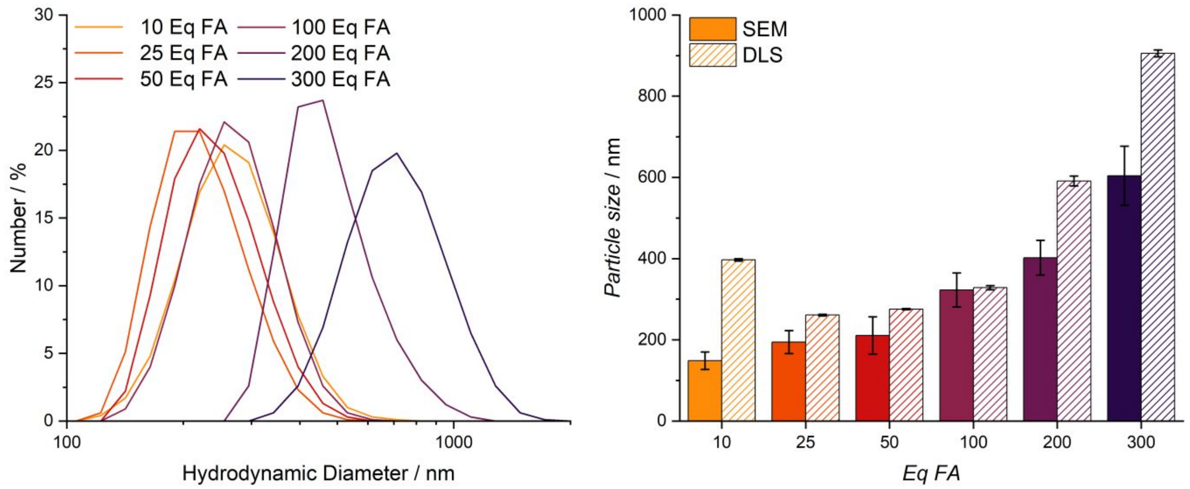

Figure S2. Left: Dynamic light scattering (DLS) measurements for the UiO-66 nanoparticles before the PSE. The values given are the average of three measurements. Right: Comparison of the particle size from SEM-images and DLS measurements.

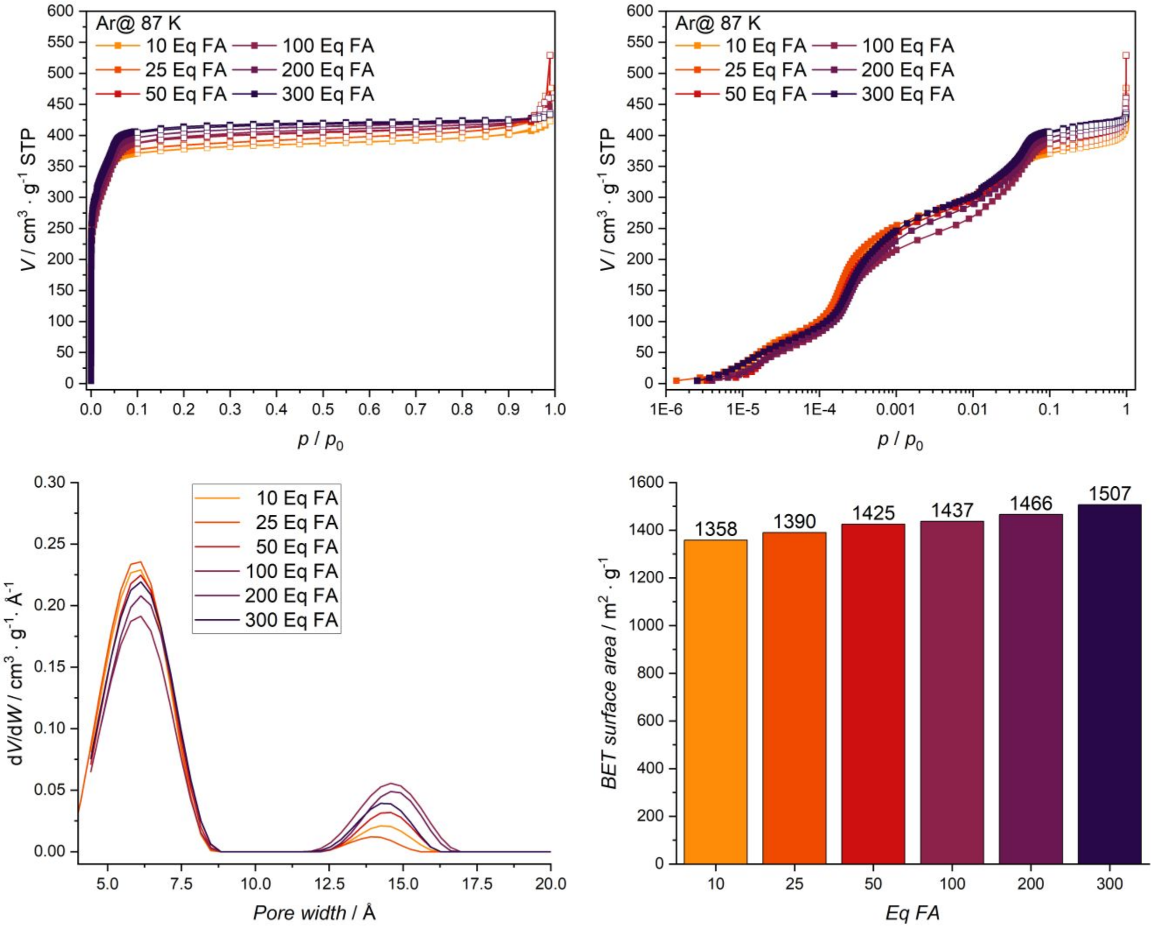

Figure S3. Argon-Sorption measurements at 87 K for the UiO-66 nanoparticles before the PSE. Top left: Argon-sorption isotherms plotted linear, top right: Argon-sorption isotherms plotted on a logarithmic scale. Full symbols for adsorption, empty symbols for desorption. Bottom left: Pore size distribution. Bottom right: BET-surface areas calculated from the argon isotherms.

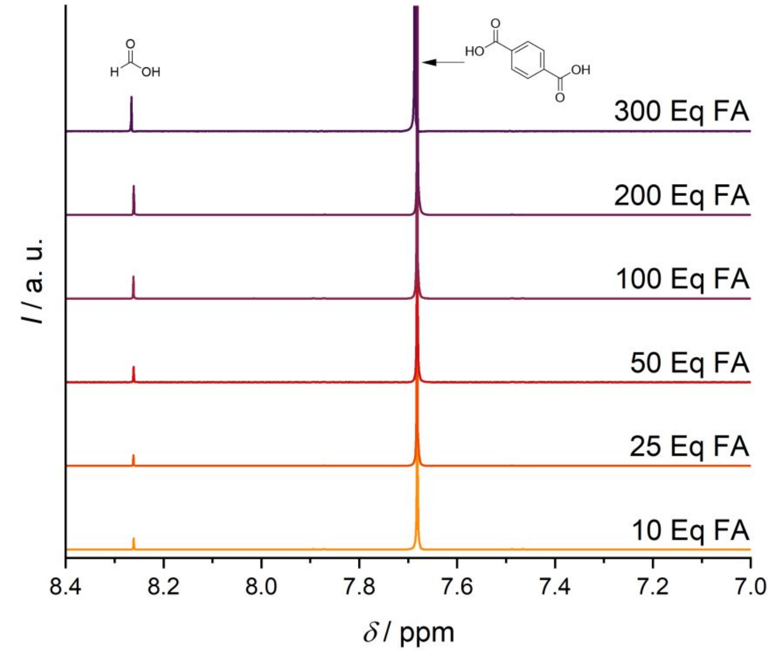

Figure S4. ^{1}H-NMR spectra after dissolution of the UiO-66 nanoparticles before the PSE. The spectra were normalized to the proton signal of H_2BDC (6.9 ppm).

Table S1. Composition of the organic phase according to the ^{1}H-NMR spectra measured after dissolution of the UiO-66 nanoparticles. The integral of H_2BDC was used as a reference and was set to 4.

Eq FA	Integral FA	Integral H_2BDC	Amount FA / %	Amount H_2BDC / %
10	0.09	4	8.26	91.74
25	0.08	4	7.41	92.59
50	0.12	4	10.71	89.29
100	0.17	4	14.53	85.47
200	0.22	4	18.03	81.97
300	0.25	4	20.00	80.00

Characterization of UiO-66 nanoparticles after the PSE

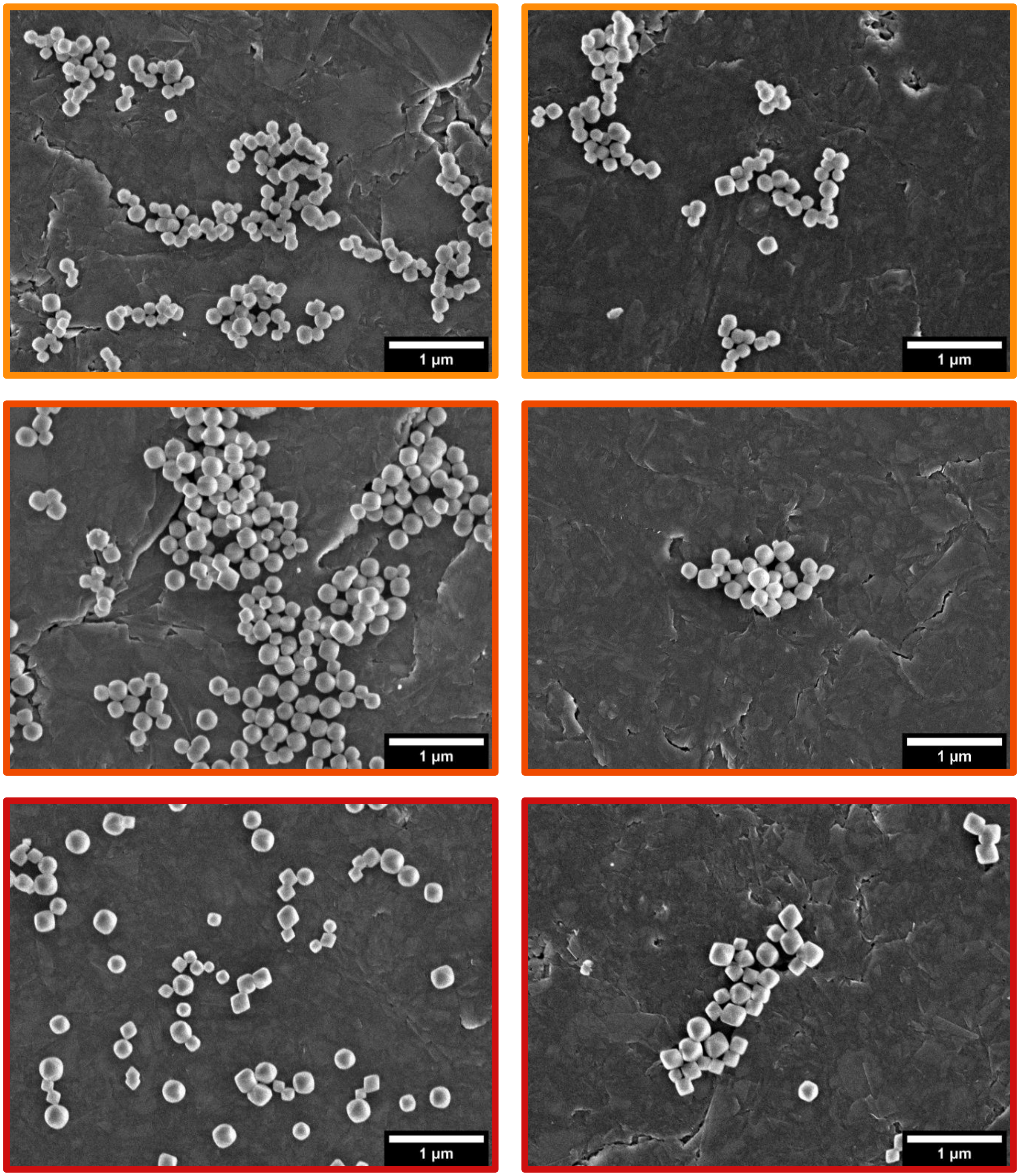

Figure S5. SEM images of UiO-66 nanoparticles before (left) and after PSE (right) Part 1. Top: 10 Eq FA, middle: 25 Eq FA, bottom: 50 Eq FA. The magnification of the SEM images is 25000x.

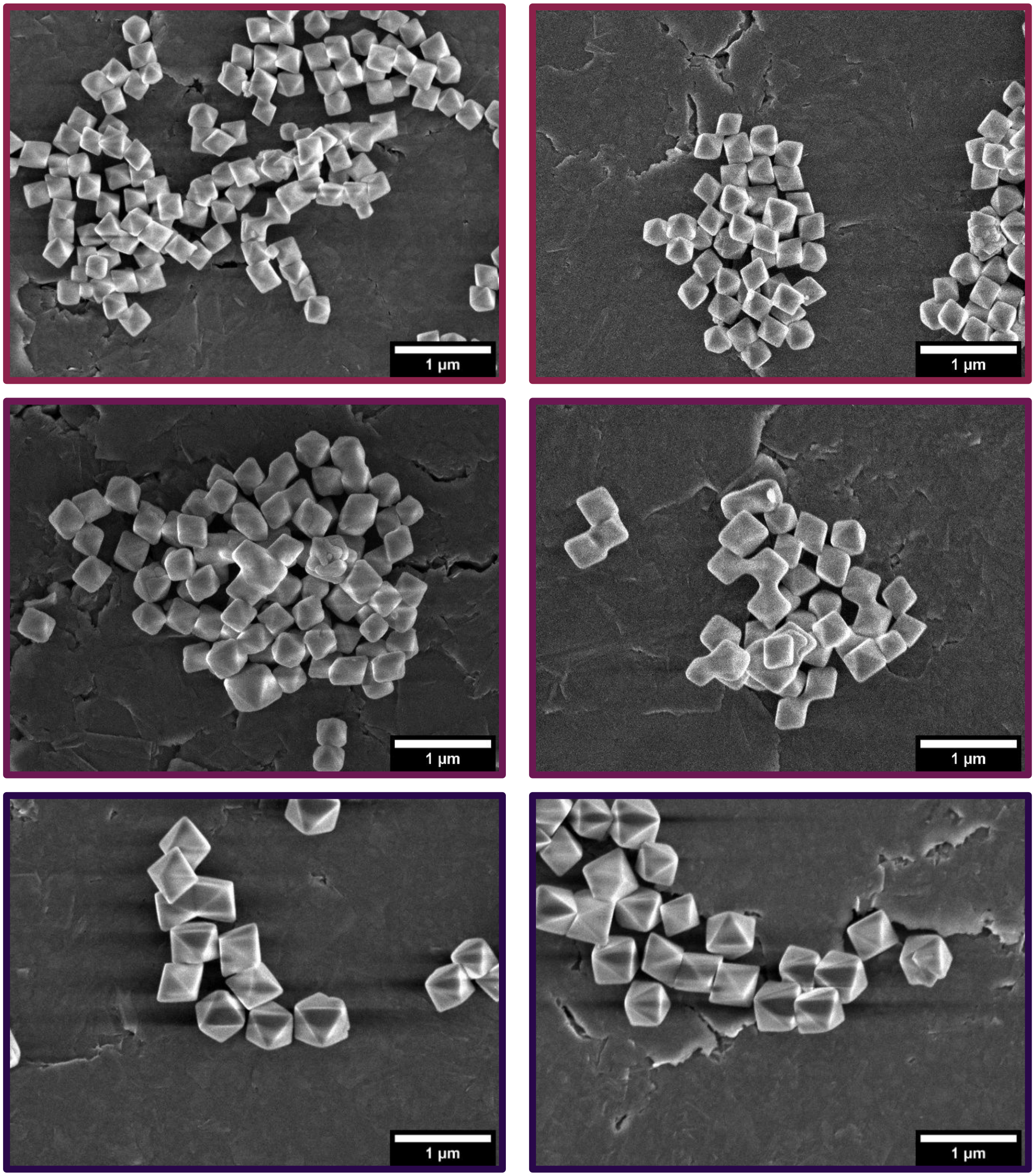

Figure S6. SEM-images of UiO-66 nanoparticles before (left) and after PSE (right) Part 2. Top: 100 Eq FA, middle: 200 Eq FA, bottom: 300 Eq FA. The magnification of the SEM images is 25000x.

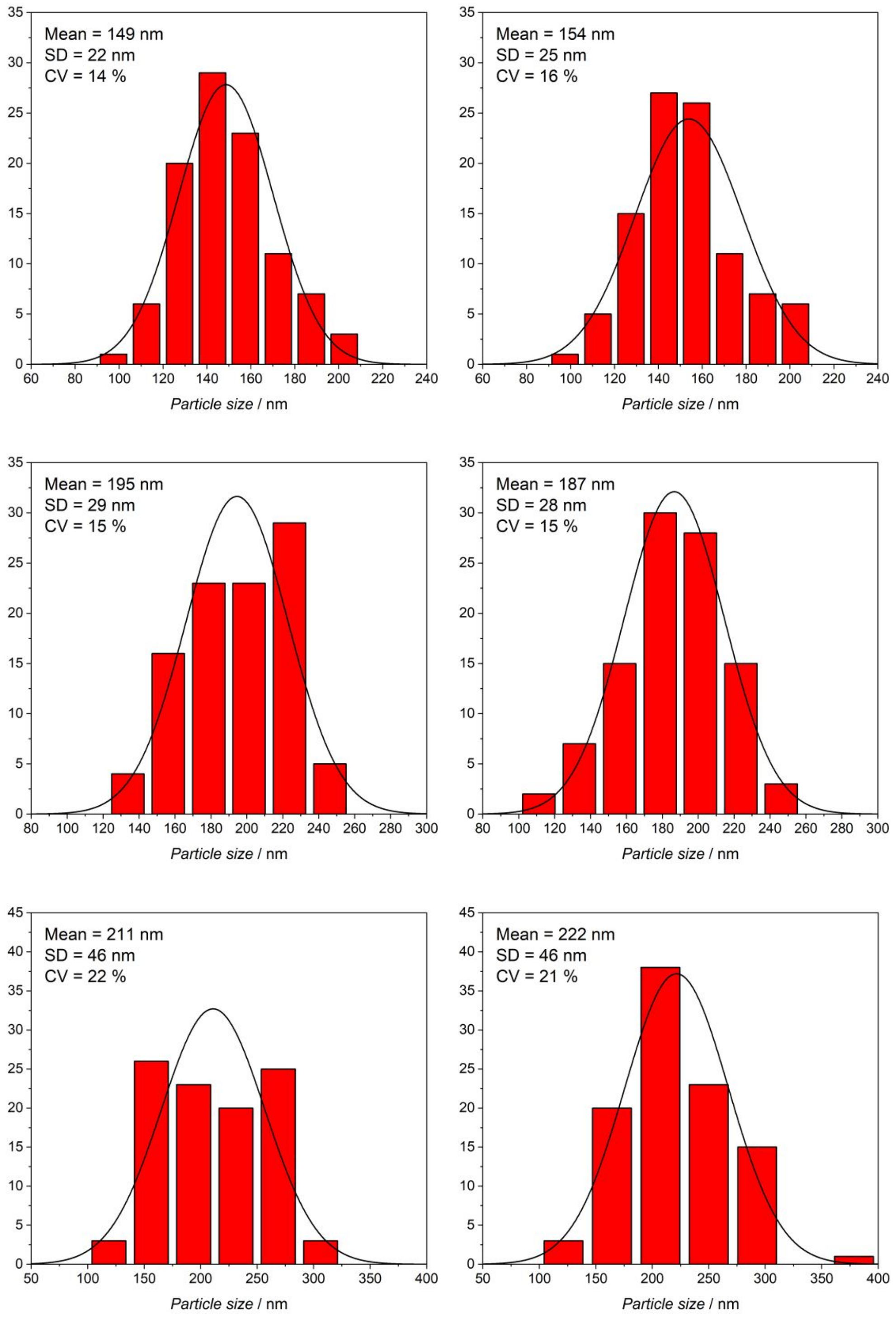

Figure S7. Particle size distribution from SEM images (100 particles measured each) of UiO-66 nanoparticles before (left) and after (right) the PSE Part 1. Top: 10 Eq FA, middle: 25 Eq FA, bottom: 50 Eq FA.

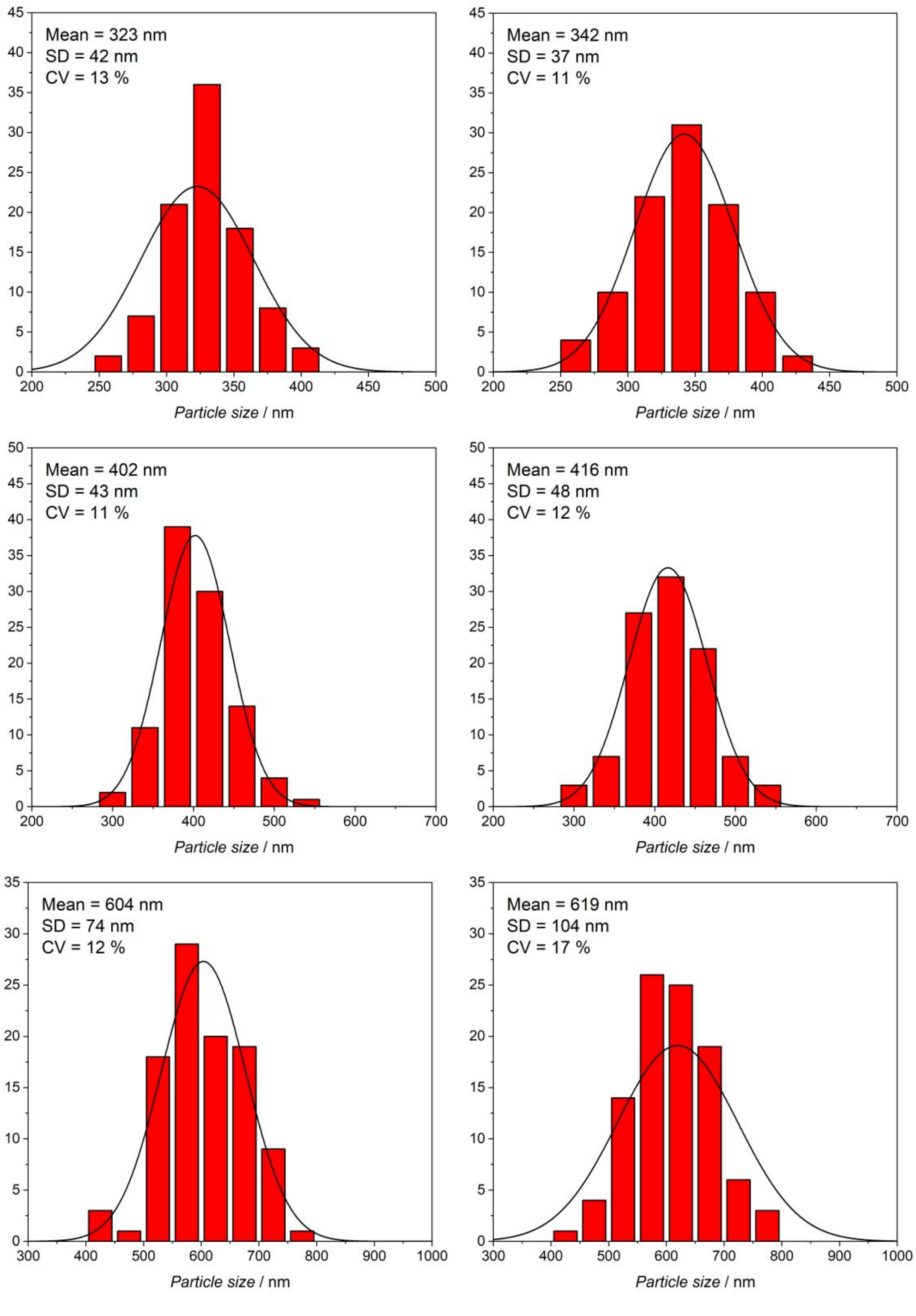

Figure S8. Particle size distribution from SEM images (100 particles measured each) of UiO-66 nanoparticles before (left) and after (right) the PSE Part 2. Top: 100 Eq FA, middle: 200 Eq FA, bottom: 300 Eq FA.

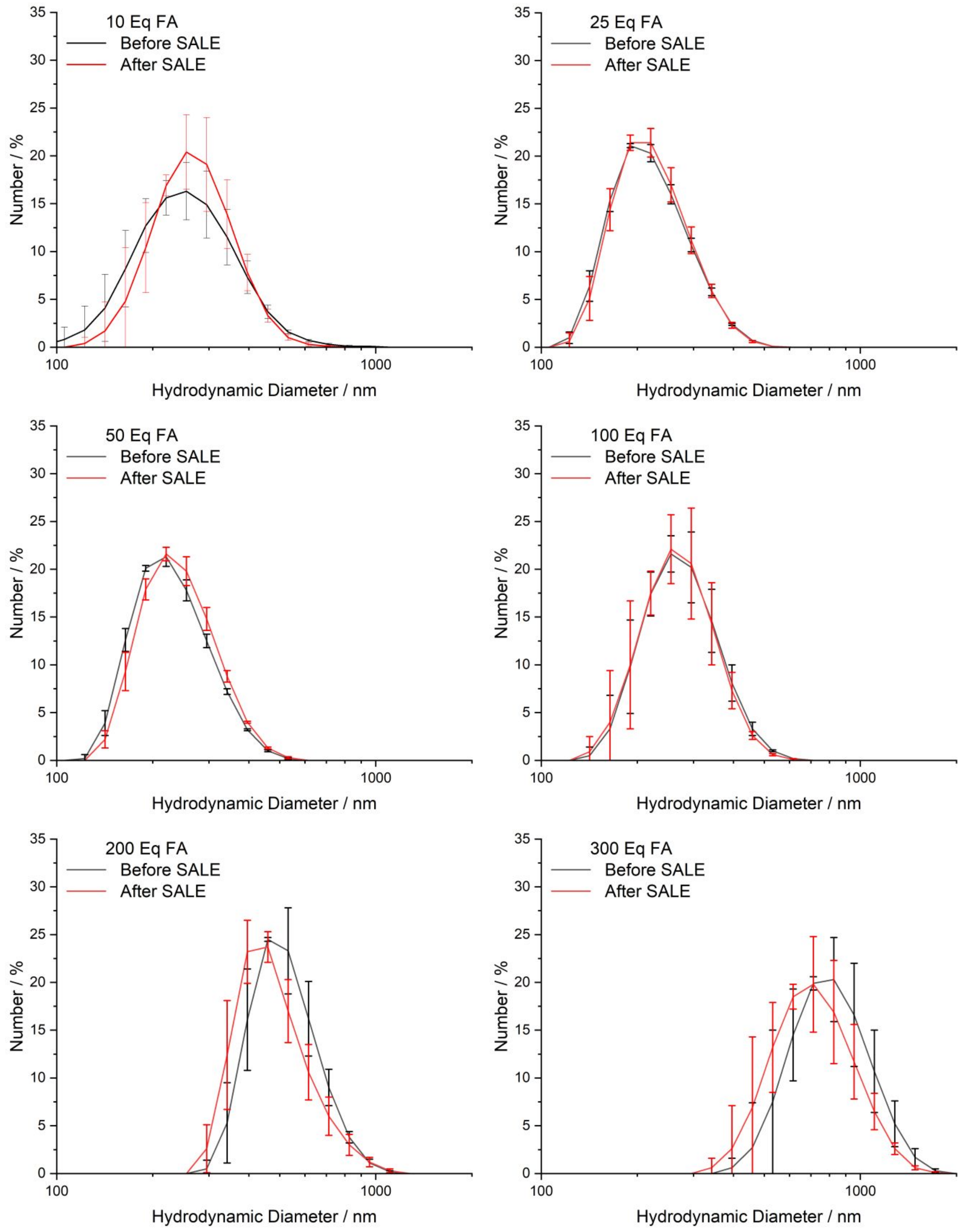

Figure S9. DLS measurements of UiO-66 nanoparticles before (black line) and after (red line) PSE. The values given are the average of three measurements.

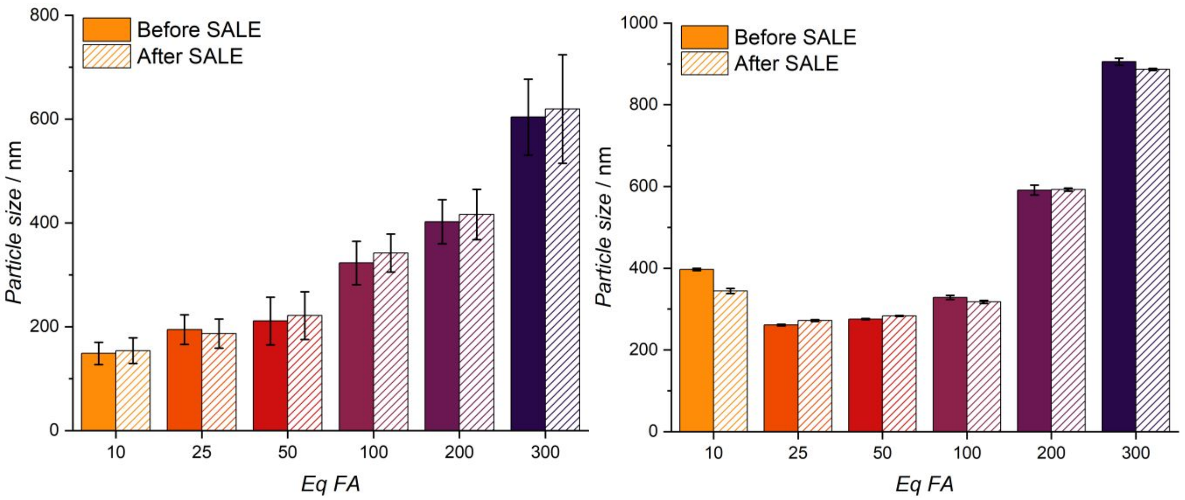

Figure S10. Comparison of the particle size before and after PSE by SEM images (left), measured from 100 particles and DLS measurements (right). The measured particle size was determined from the mean value of three DLS measurements.

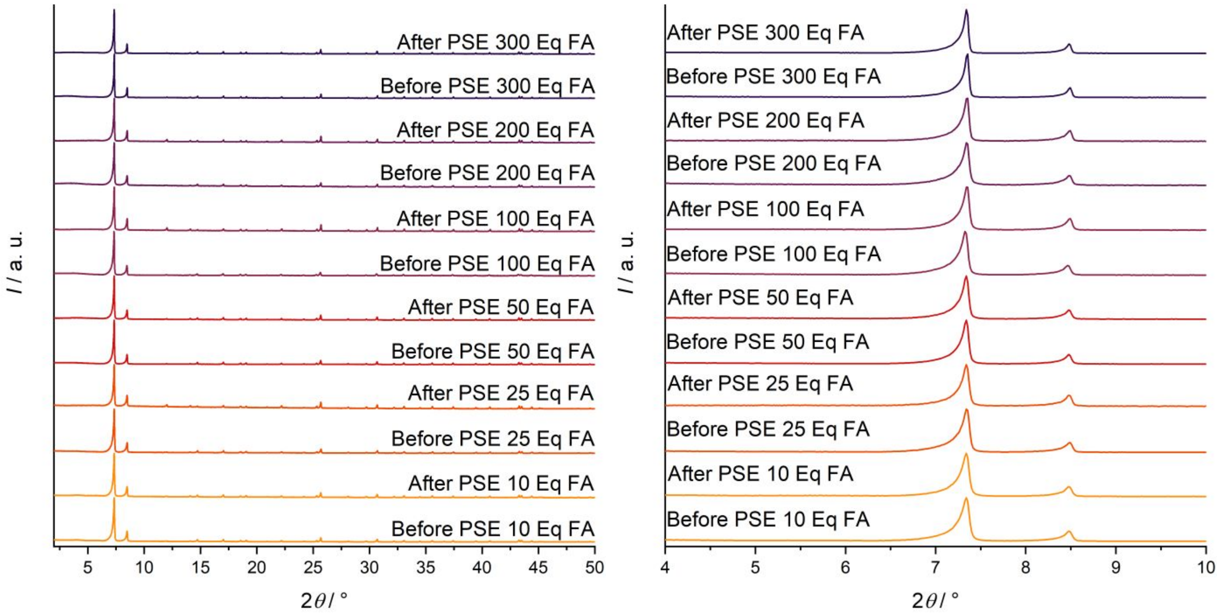

Figure S11. Comparison of the PXRDs before and after PSE. Left: PXRDs from 2 – 50° 2θ. Right: PXRDs from 4 – 10° 2θ.

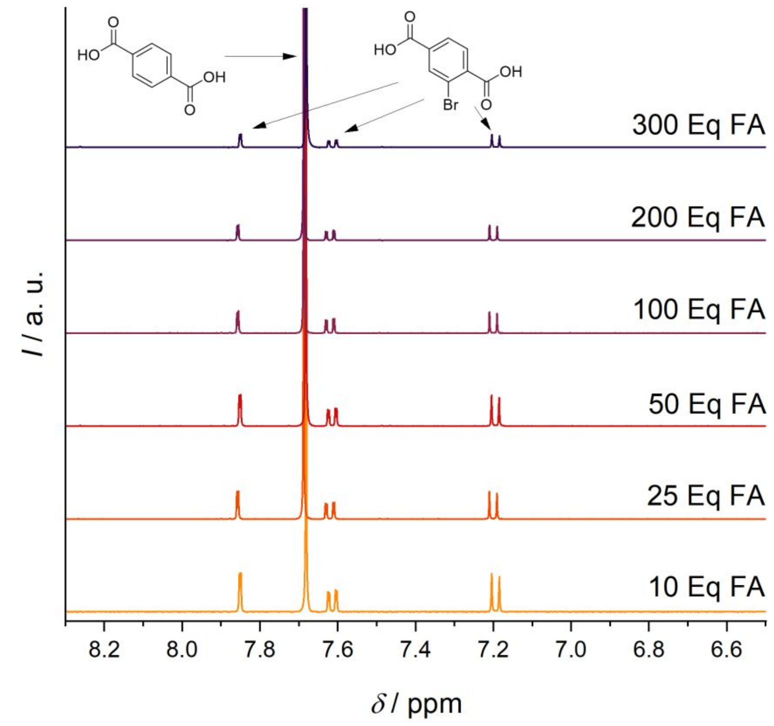

Figure S12. ^{1}H-NMR spectra after dissolution of the UiO-66 nanoparticles before the PSE. The spectra were normalized to the proton signal of H_2BDC (6.9 ppm). The formic acid peak (8.27 ppm) has disappeared after the PSE.

Table S2. Composition of the organic part according to the ^{1}H-NMR spectra measured after dissolution of the UiO-66 nanoparticles after the PSE. The integral of H_2BDC was used as a reference and was set to 4. The integral of H_2BDC-Br shows the combined integral of the three protons.

Eq FA	Integral H_2BDC-Br	Integral H_2BDC	Amount H_2BDC-Br / %	Amount H_2BDC / %
10	2.20	4	42.31	57.69
25	1.76	4	36.97	63.03
50	1.78	4	37.24	62.76
100	1.51	4	33.48	66.52
200	1.00	4	25.00	75.00
300	0.72	4	19.35	80.65

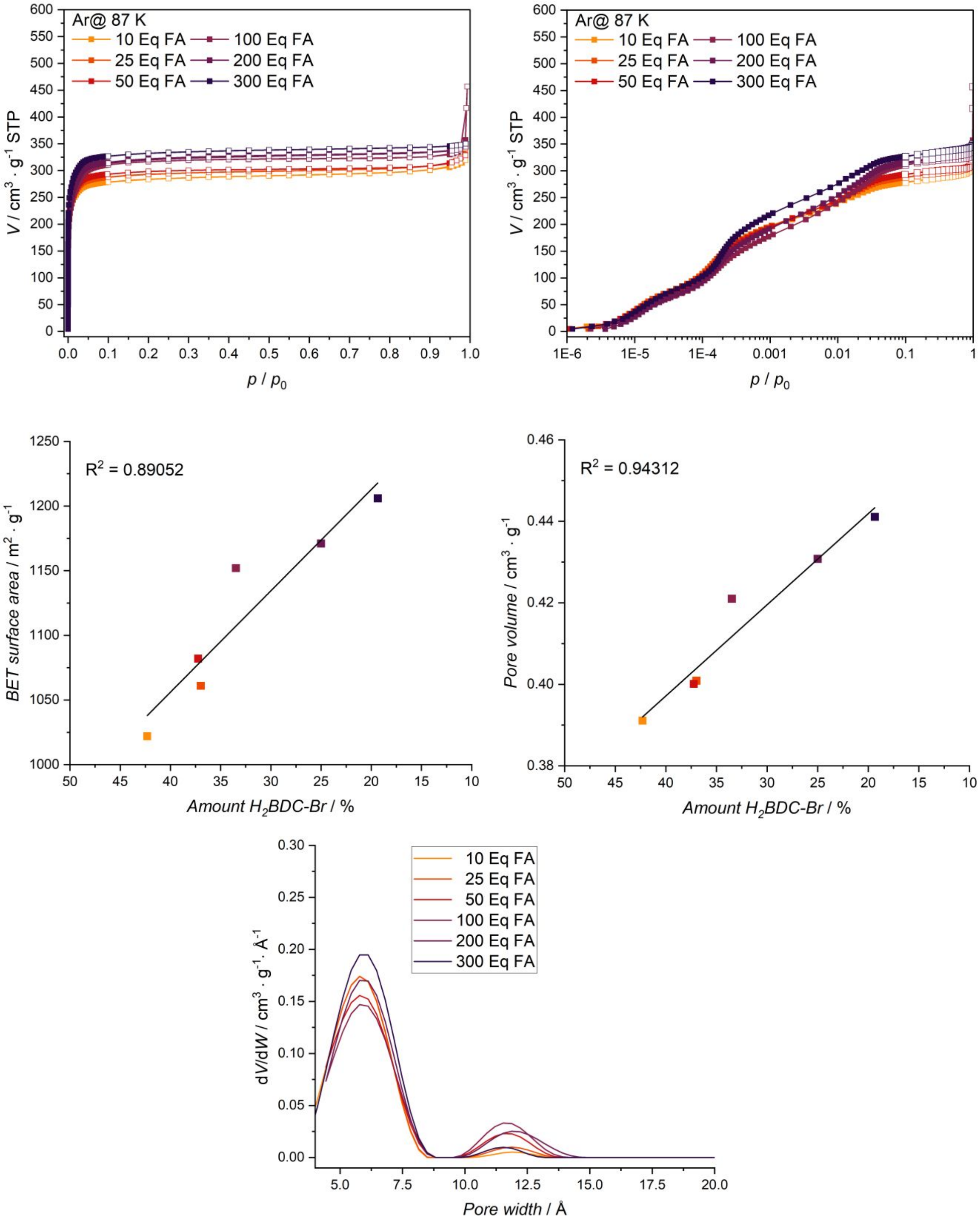

Figure S13. Argon-Sorption measurements at 87 K for the UiO-66 nanoparticles after the PSE. Top left: Argon-sorption isotherms plotted linear, top right: Argon-sorption isotherms plotted on a logarithmic scale. Full symbols for adsorption, empty symbols for desorption. Middle: Plot of the BET-surface area (middle) and pore volume (right) versus the amount of incorporated linker H_2BDC-Br measured from ^{1}H-NMR dissolution experiments. Bottom: Pore size distribution calculated from argon isotherms.

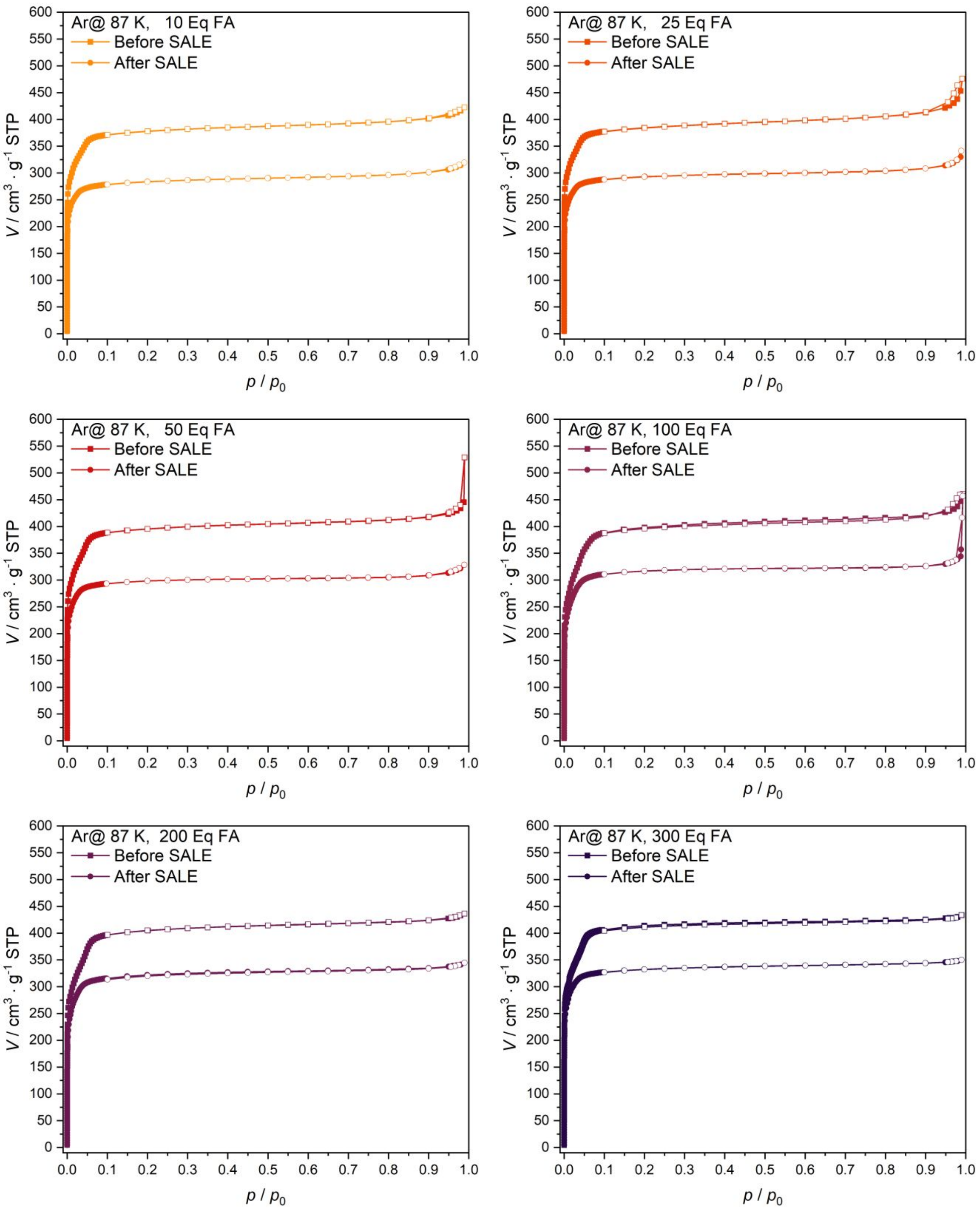

Figure S14. Comparison of argon-sorption isotherms before (squares) and after (circles) PSE. Full symbols for adsorption, empty symbols for desorption.

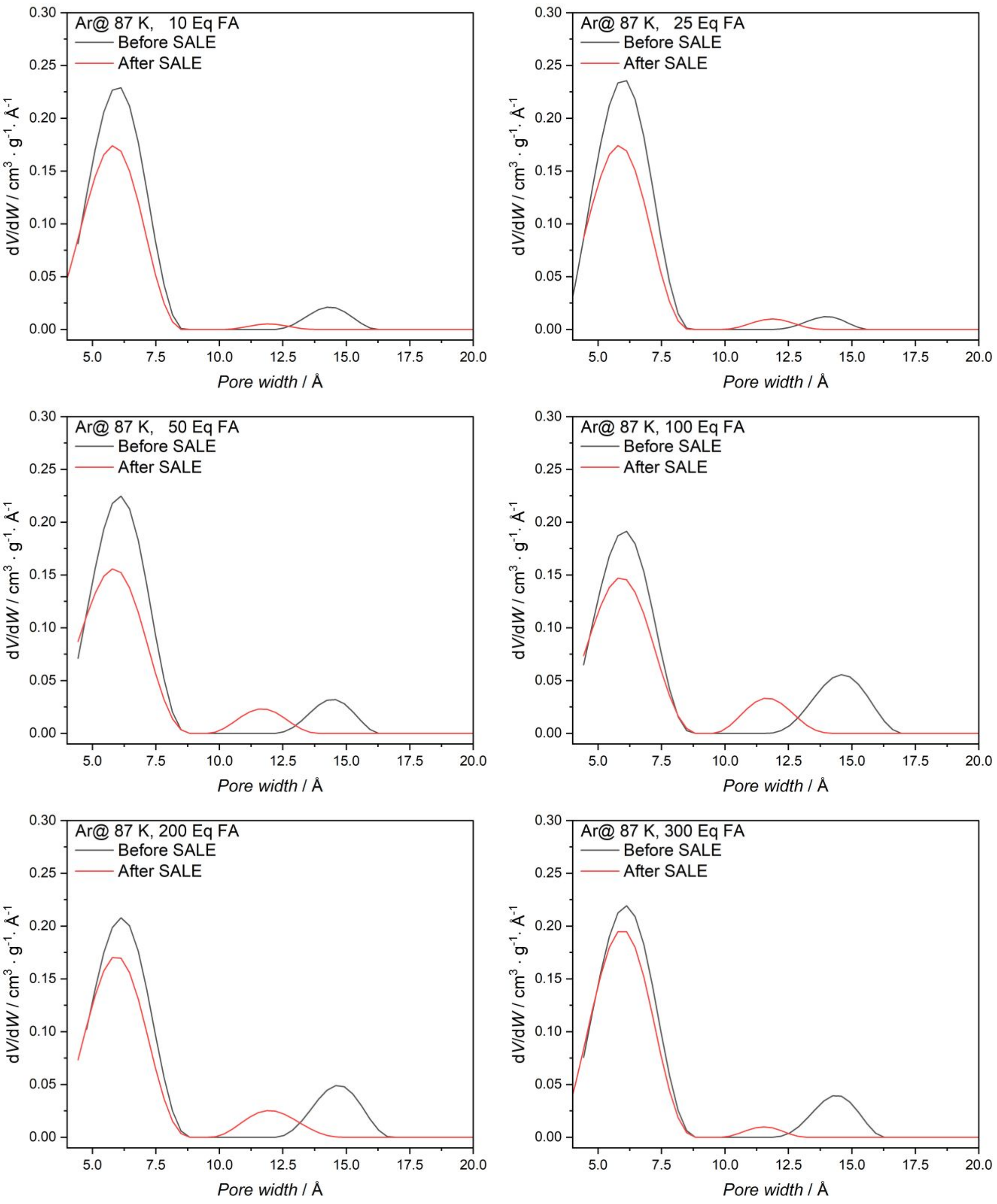

Figure S15. Comparison of pore size distributions calculated from argon-sorption isotherms at 87 K before (black) and after (red) PSE.

Section 3 Kinetic investigation of linker insertion versus linker exchange

PSE on FA-containing UiO-66 nanoparticles

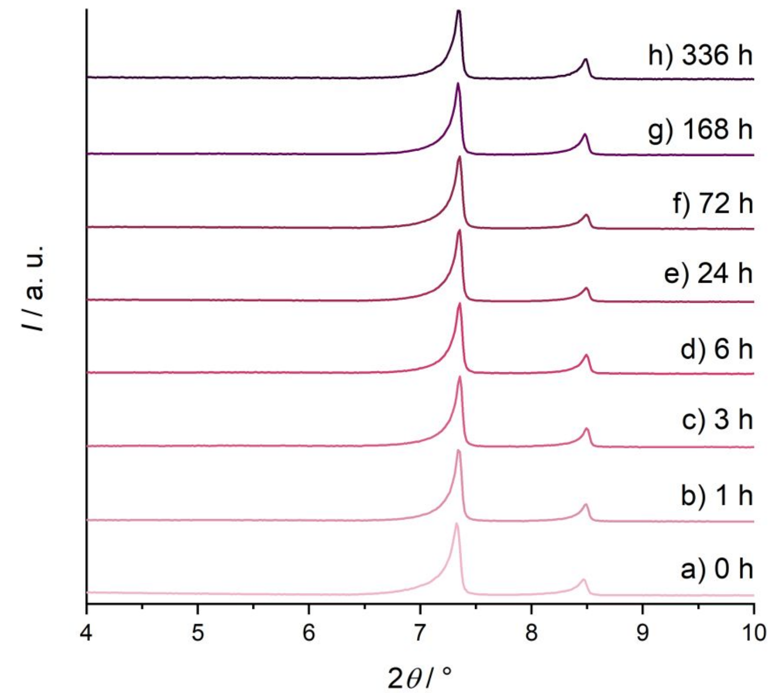

Figure S16. PXRDs from 4 – 10° 2θ after PSE reaction time of a) 0 h, b) 1 h, c) 3 h, d) 6 h, e) 24 h, f) 72 h, g) 168 h, h) 336 h.

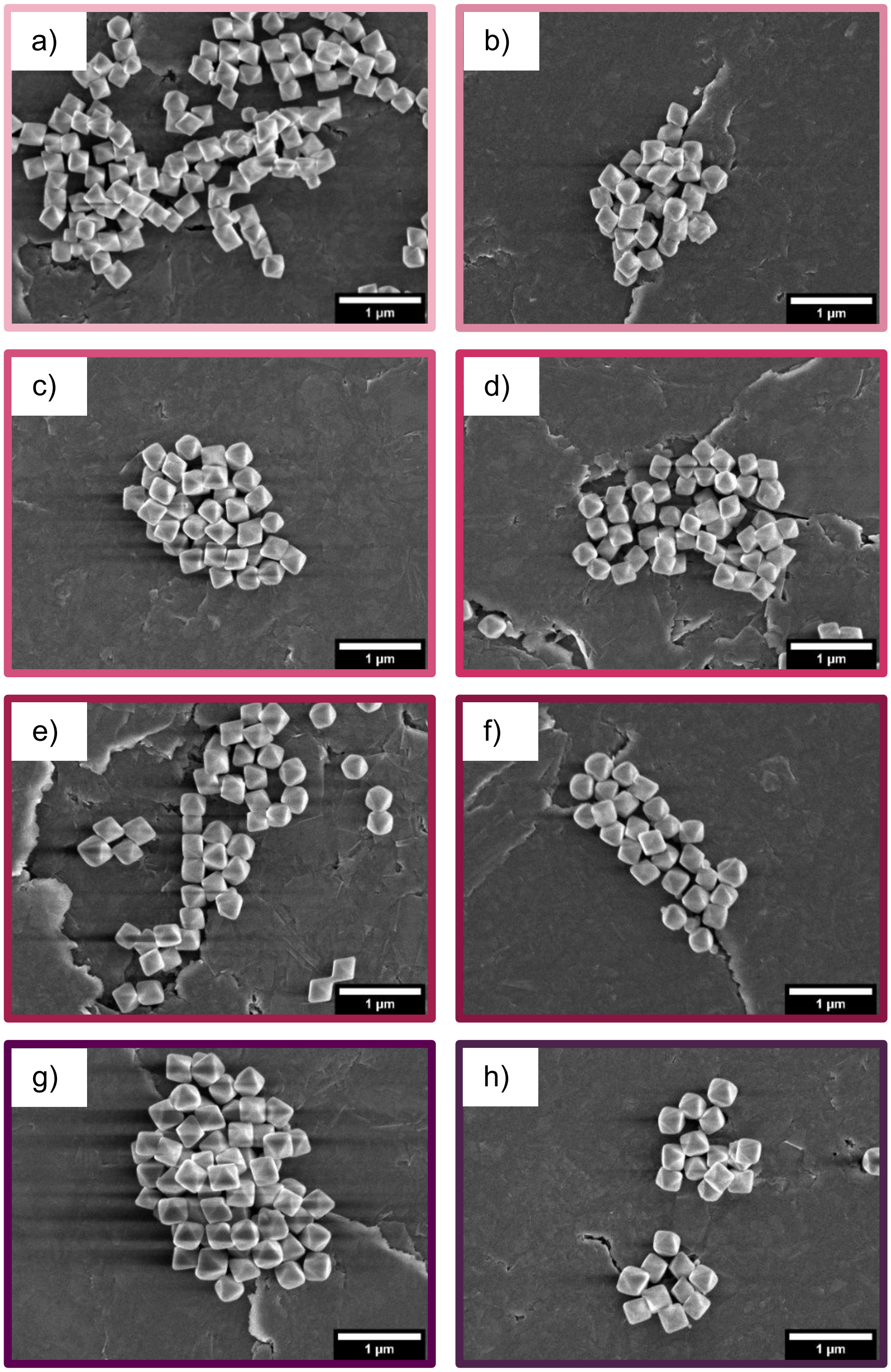

Figure S17. SEM images after PSE reaction time of a) 0 h, b) 1 h, c) 3 h, d) 6 h, e) 24 h, f) 72 h, g) 168 h, h) 336 h. The magnification of the SEM images is 25000x.

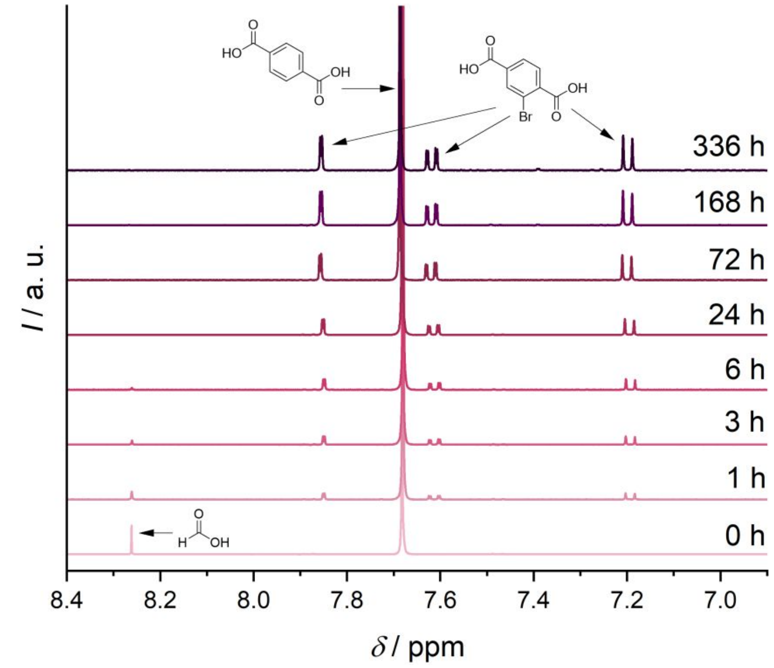

Figure S18. ^{1}H-NMR spectra after dissolution of the UiO-66 nanoparticles after the PSE. The spectra were normalized to the proton signal of H_2BDC (6.9 ppm).

Table S3. Composition of the organic part according to the ^{1}H-NMR spectra measured after dissolution of the UiO-66 nanoparticles after the PSE. The integral of H_2BDC was used as a reference and was set to 4. The integral of H_2BDC-Br shows the combined integral of the three protons.

t / h	Integral FA	Integral H_2BDC-Br	Integral H_2BDC	Amount FA / %	Amount H_2BDC-Br / %	Amount H_2BDC / %
0	0.17	0.00	4	14.53	0.00	85.47
1	0.07	0.32	4	5.95	9.07	84.99
3	0.04	0.44	4	3.37	12.36	84.27
6	0.02	0.58	4	1.65	15.93	82.42
24	0.00	0.90	4	0.00	23.08	76.92
72	0.00	1.51	4	0.00	33.48	66.52
168	0.00	1.82	4	0.00	37.76	62.24
336	0.00	1.95	4	0.00	39.39	60.61

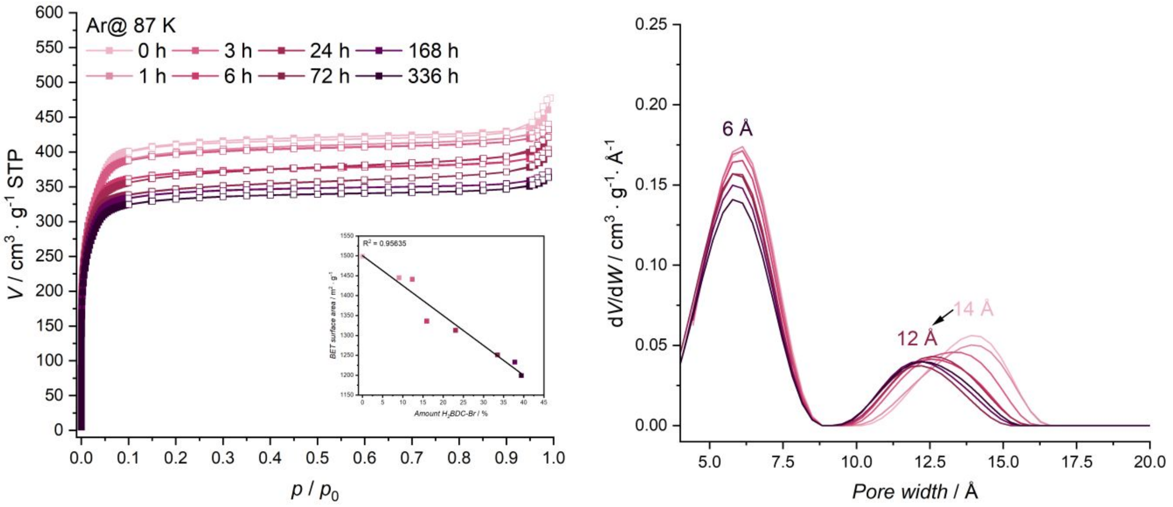

Figure S19. Argon-sorption measurements at 87 K of UiO-66 nanoparticles after PSE with varying reaction time. Left: Argon-sorption isotherms with the inset of the BET-surface area plotted against the amount of incorporated linker measured via ^{1}H-NMR dissolution experiments. Right: Pore size distribution calculated from argon-sorption measurements.

PSE with H_2BDC-H to synthesize FA-free UiO-66 nanoparticles with H_2BDC-H

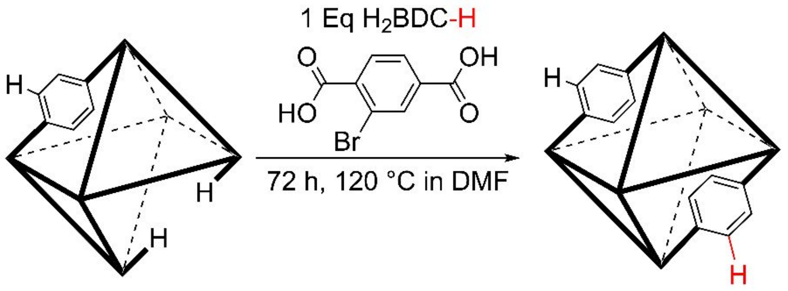

Figure S20. Schematic PSE conditions for the synthesis of FA-free UiO-66. The synthetic procedure is given under PSE on UiO-66 nanoparticles with H_2BDC (UiO-66-H_H) in section 1.

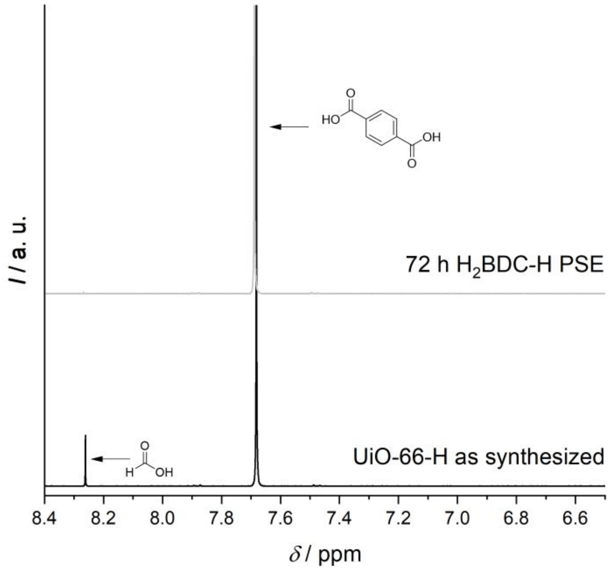

Figure S21. ^{1}H-NMR spectra after dissolution of the UiO-66 nanoparticles after the PSE with H_2BDC-H. The spectra were normalized to the proton signal of H_2BDC (6.9 ppm). The formic acid was completely removed during the PSE procedure.

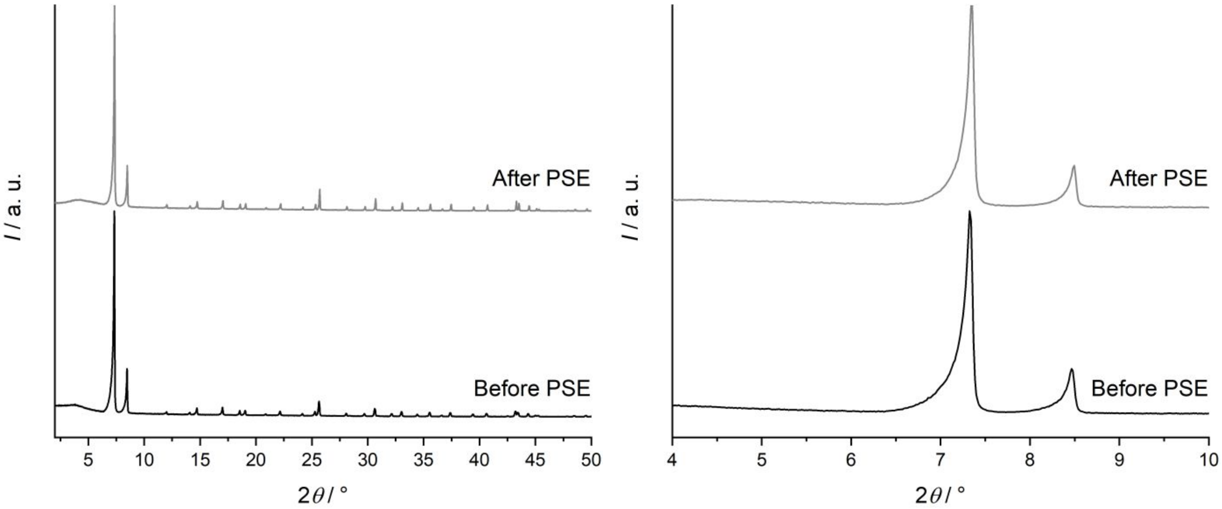

Figure S22. Comparison of the PXRDs before and after the PSE with H_2BDC-H on UiO-66. Left: PXRDs from 2 – 50° 2θ. Right: PXRDs from 4 – 10° 2θ.

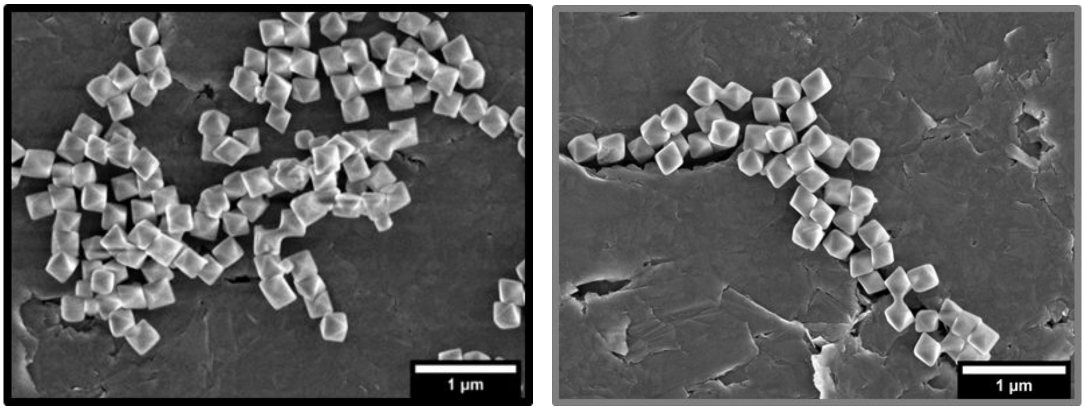

Figure S23. SEM images of UiO-66 nanoparticles before (left) and after (right) PSE with H_2BDC. The magnification of the SEM-images is 25000x.

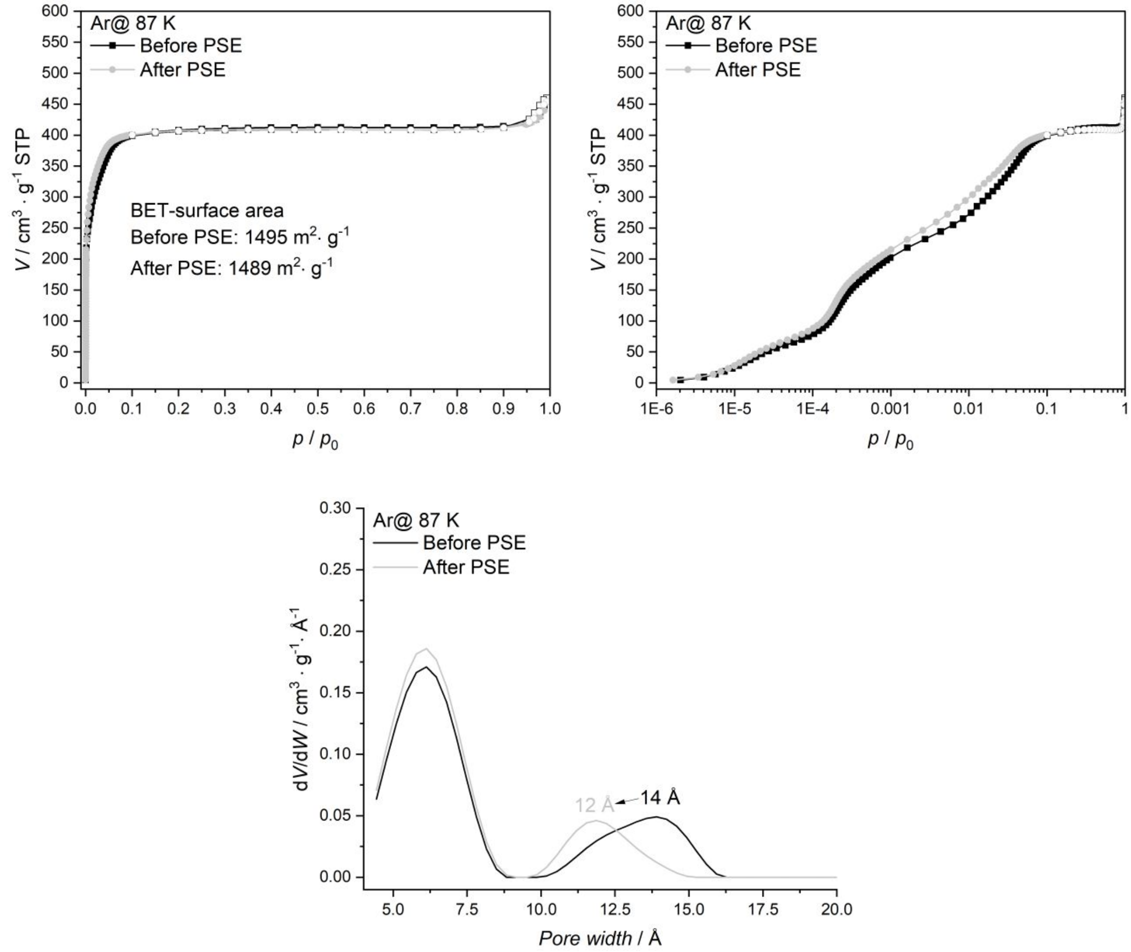

Figure S24. Argon-sorption measurements at 87 K on UiO-66 nanoparticles after PSE with H_2BDC-H. Top left: Argon-sorption isotherms plotted linear, top right: Argon-sorption isotherms plotted on a logarithmic scale. Bottom: Pore size distribution calculated from argon-sorption isotherms.

PSE on UiO-66 nanoparticles without formic acid

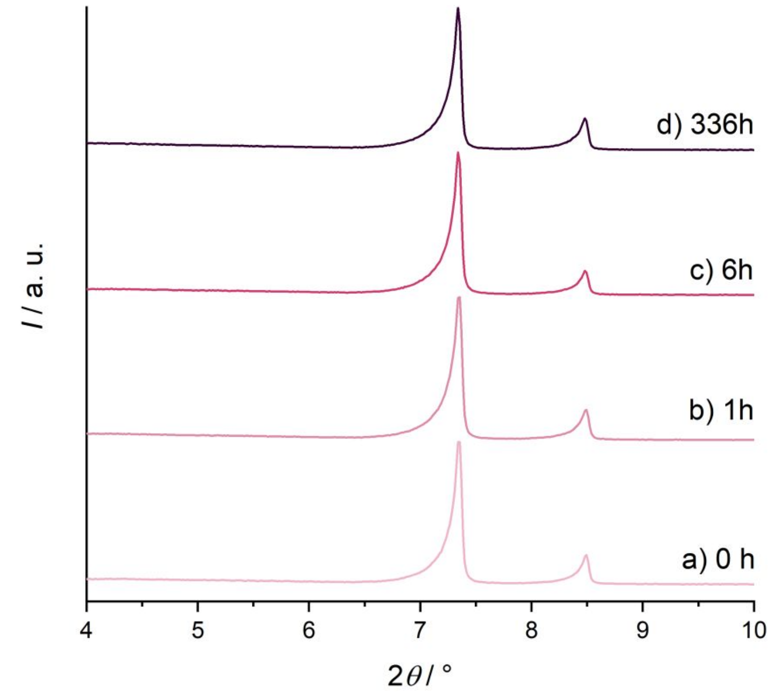

Figure S25. PXRDs from 4 – 10° 2θ of formic acid-free UiO-66 nanoparticles after PSE with H_2BDC-Br for a) 0 h, b) 1 h, c) 6 h, d) 336 h.

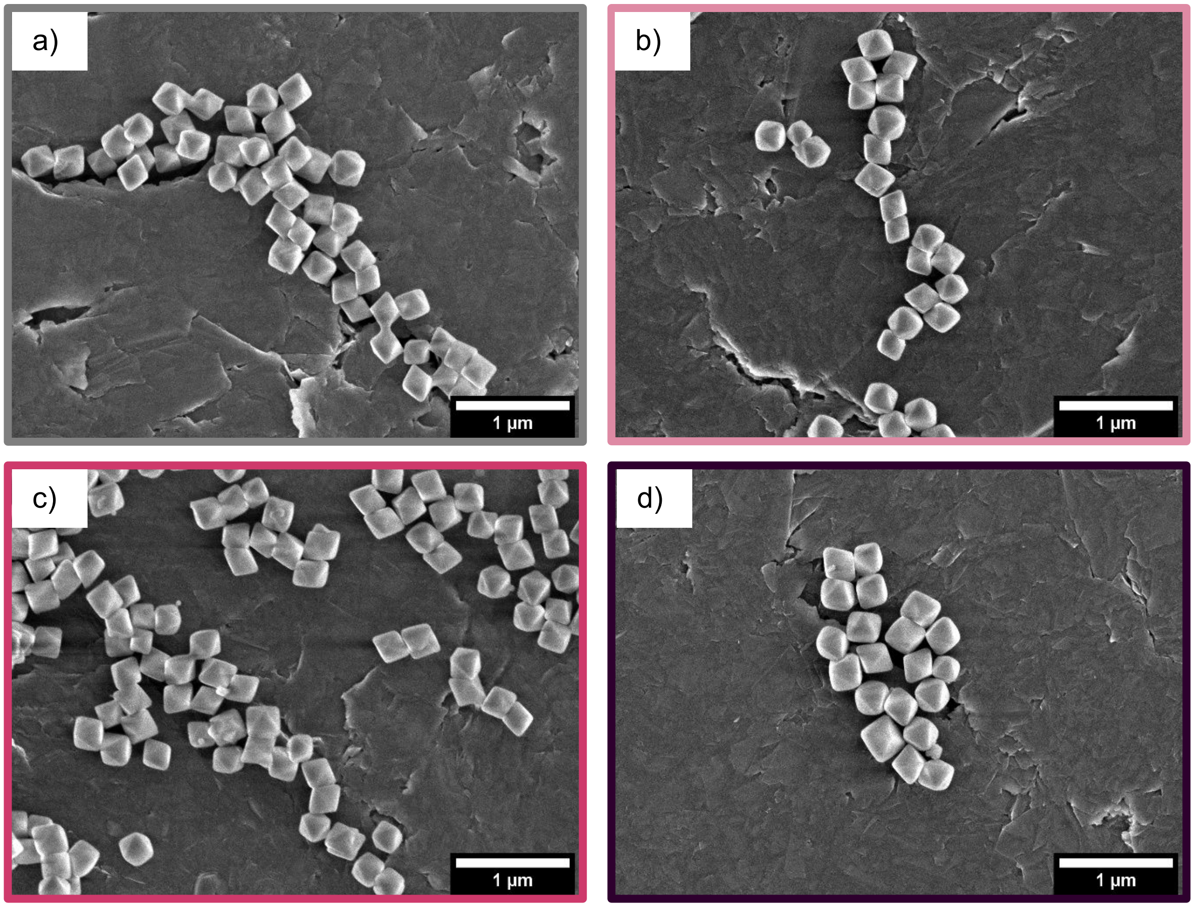

Figure S26. SEM images of formic acid-free UiO-66 nanoparticles after PSE with H_2BDC-Br for a) 0 h, b) 1 h, c) 6 h, d) 336 h. The magnification of the SEM images is 25000x.

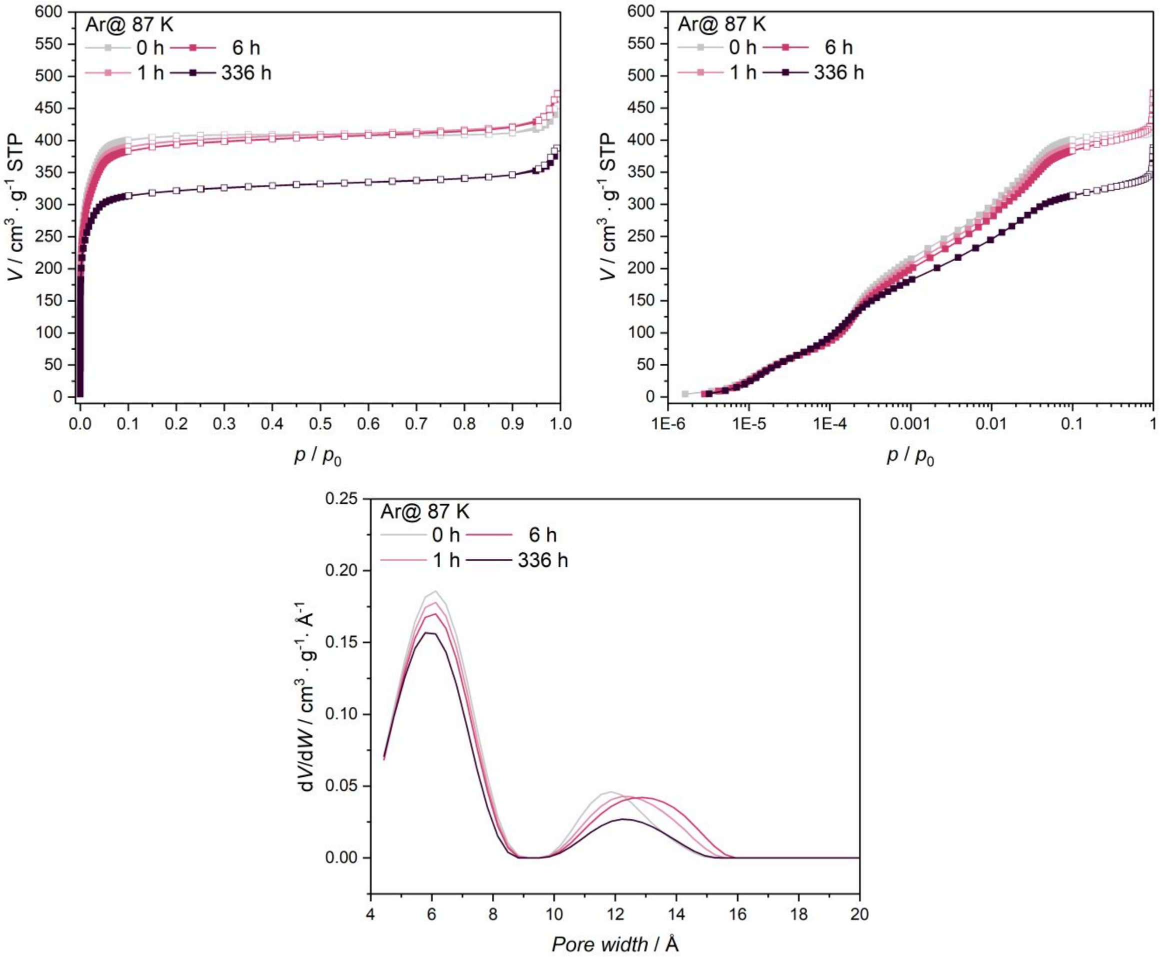

Figure S27. Argon-sorption measurements at 87 K on UiO-66 nanoparticles without formic acid after PSE with H_2BDC-Br for varying reaction times. Top left: Argon-sorption isotherms plotted linear, top right: Argon-sorption isotherms plotted on a logarithmic scale. Bottom: Pore size distribution calculated from argon-sorption isotherms.

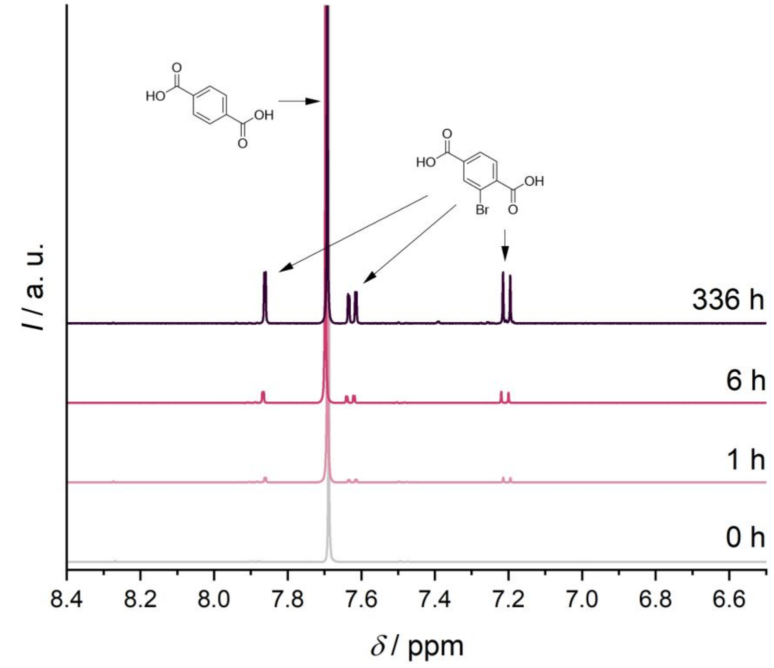

Figure S28. ^{1}H-NMR spectra after dissolution of formic acid-free UiO-66 nanoparticles after the PSE with H_2BDC-Br for a specific amount of time. The spectra were normalized to the proton signal of H_2BDC (6.9 ppm).

Table S4. Composition of the organic part according to the ^{1}H-NMR spectra measured after dissolution of the formic acid-free UiO-66 nanoparticles after the PSE with H_2BDC-Br. The integral of H_2BDC was used as a reference and was set to 4. The integral of H_2BDC-Br shows the combined integral of the three protons.

t **/ h**	**Integral FA**	**Integral H_2BDC-Br**	**Integral H_2BDC**	**Amount FA / %**	**Amount H_2BDC-Br / %**	**Amount H_2BDC / %**
0	0.00	0.00	4	0.00	0.00	100.00
1	0.00	0.16	4	0.00	5.06	94.94
6	0.00	0.42	4	0.00	12.28	87.72
336	0.00	1.92	4	0.00	39.02	60.98

Section 4 Calculating the minimum incorporation depth of UiO-66 core-shell nanoparticles

Calculation of the minimum incorporation depth

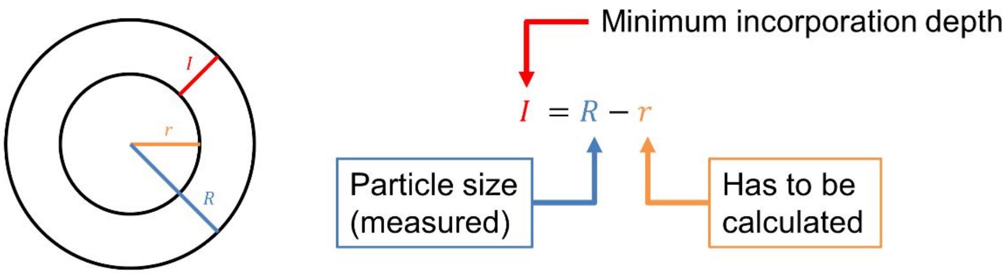

Figure S29. Geometrical model for the calculation of the minimum incorporation depth using a spherical shell.

For the calculation of the minimum incorporation depth *(I)* using a spherical shell model (**Figure S29**), it is essential to know the particle size of the core-shell particle (R), which can routinely be measured using TEM and SEM. The core radius (r) is then calculated under the assumption of a complete exchange in the shell, meaning the shell consists solely of the newly incorporated linker after the PSE.

This assumption allows us too equate the ratio of the volumes (V_I/V_R) with the proportion of the newly incorporated linker after the PSE ($\text{Linker}_{\text{PSE}}$) leading to

$$[V_I/V_R] = \text{Linker}_{\text{PSE}} \tag{S1}$$

The amount of newly incorporated linker can be easily accessed by ^{1}H-NMR digestion experiments. Under this assumption, the volume of the shell V_I can be calculated by

$$V_I = [V_I/V_R] \cdot V_R = \text{Linker}_{\text{PSE}} \cdot V_R \tag{S2}$$

With the calculated volume V_I and the volume V_R, which was measured using SEM or TEM, the volume of the core can be measured with the following equation

$$V_R = V_I + V_r \rightarrow V_r = V_R - V_I \tag{S3}$$

Further, the volume of the core V_r can be measured by using the equation of the volume for a sphere

$$r = \sqrt[3]{\frac{V_r \cdot 3}{\pi \cdot 4}} \tag{S4}$$

At last, the minimum incorporation depth can be calculated by

$$I = R - r \tag{S5}$$

With this method, the minimum incorporation depth was calculated for the UiO-66 core-shell nanoparticles presented in this work. The summary of the minimum incorporation depth and the measured values for this calculation are summarized in **Table S5**.

Table S5. The minimum incorporation depth for the UiO-66 core-shell nanoparticles in this work. The first symbol of UiO-66-X-Y describes which linker is located in the core.

Sample	Particle size SEM / nm V_R	$Linker_{PSE}$ NMR $[V_p/V_R]$	V_r / nm^3	r / nm	I / nm
UiO-66-H_Br_10 Eq FA	148.7	42.31	728368	61.9	12.5
UiO-66-H_Br_25 Eq FA	194.5	36.97	1424502	83.4	13.9
UiO-66-H_Br_50 Eq FA	211.0	37.24	1831631	90.3	15.2
UiO-66-H_Br_100 Eq FA	322.8	33.48	5896571	140.9	20.5
UiO-66-H_Br_200 Eq FA	402.1	25.00	8510221	182.7	18.4
UiO-66-H_Br_300 Eq FA	603.9	19.35	22319440	281.1	20.9
UiO-66-H_Br_1 h	322.8	9,07	1596521	156.4	5.0
UiO-66-H_Br_3 h	322.8	12.36	2176716	154.5	6.9
UiO-66-H_Br_6 h	322.8	15.93	2806247	152.3	9.1
UiO-66-H_Br_24 h	322.8	23.08	4064218	147.9	13.5
UiO-66-H_Br_72 h	322.8	33.48	5896571	140.9	20.5
UiO-66-H_Br_168 h	322.8	37.76	6650030	137.8	23.6
UiO-66-H_Br_336 h	322.8	39.39	6937909	136.6	24.8
UiO-66-Br_H	539.0	39.16	32107582	228.4	41.1

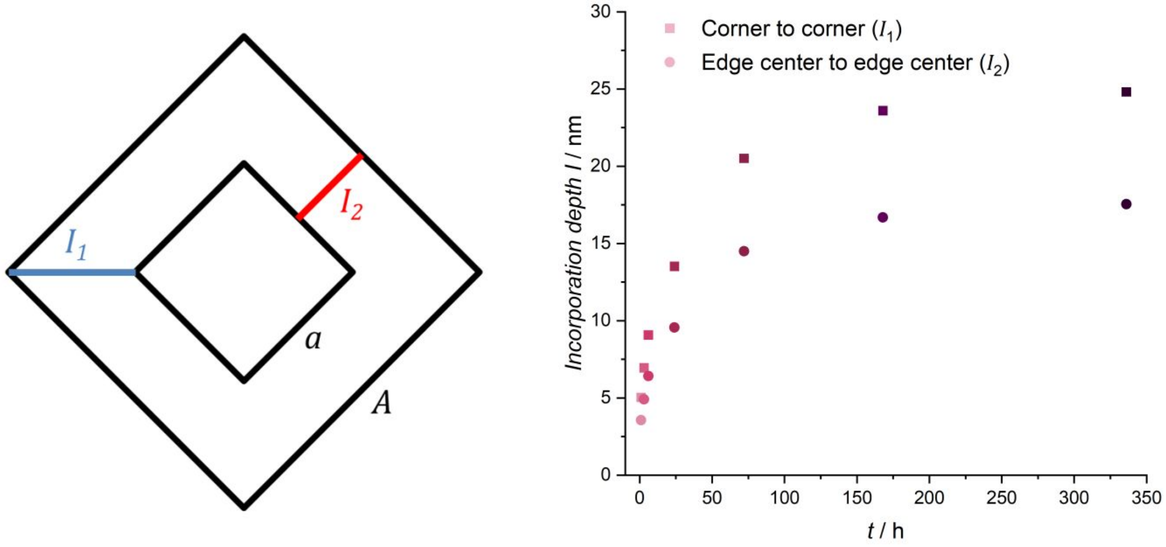

Figure S30. Geometrical model for the calculation of the minimum incorporation depth using an octahedral shell. Left: 2D schematic of an octahedral particle with an octahedral shell. a represents the edge length of the octahedron in the core and A the edge length of the shell. The incorporation depth can be defined with I_1, ranging from corner to corner, and I_2, ranging from the center of one edge of the core to the center of an edge of the shell. Right: Plot of the minimum incorporation depths calculated from the time-dependent investigation against the time of exchange. The trend is consistent with the spherical shell model, with lower minimum incorporation depths calculated when using the definition of I_2 and identical for the definition of I_1.

Characterization of UiO-66-Br_H core-shell nanoparticles

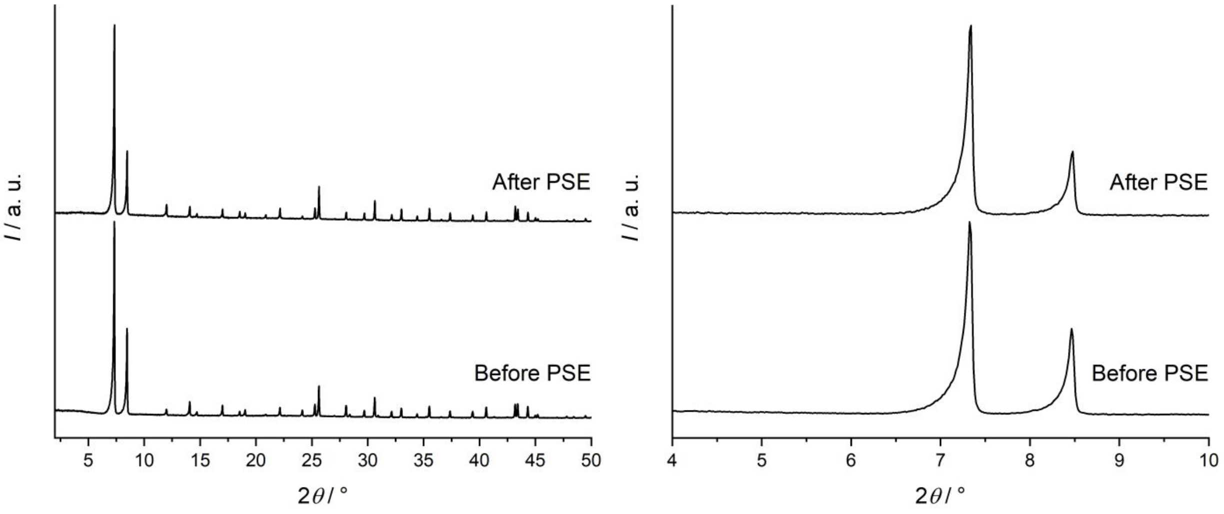

Figure S31. PXRD measurements of the UiO-66-Br nanoparticles before and after PSE. Left: PXRDs from 2 – 50° 2θ. Right: PXRDs from 4 – 10° 2θ.

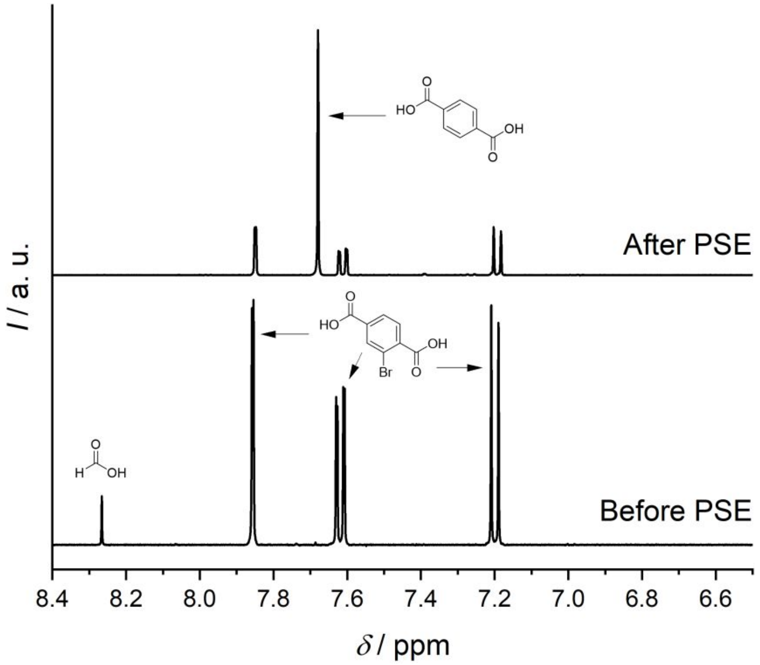

Figure S32. ^{1}H-NMR measurements after dissolution of the UiO-66-Br nanoparticles before and after PSE.

Table S6. Composition of the organic part according to the ^{1}H-NMR spectra measured after dissolution of the UiO-66-Br nanoparticles after the PSE with H_2BDC. The integral of H_2BDC was used as a reference and was set to 4. The integral of H_2BDC-Br shows the combined integral of the three protons.

Sample	Integral FA	Integral H_2BDC-Br	Integral H_2BDC	Amount FA / %	Amount H_2BDC-Br / %	Amount H_2BDC / %
Before PSE	0.08	3.01	0	7.38	92.62	0.00
After PSE	0.00	4.66	4	0.00	60.84	39.16

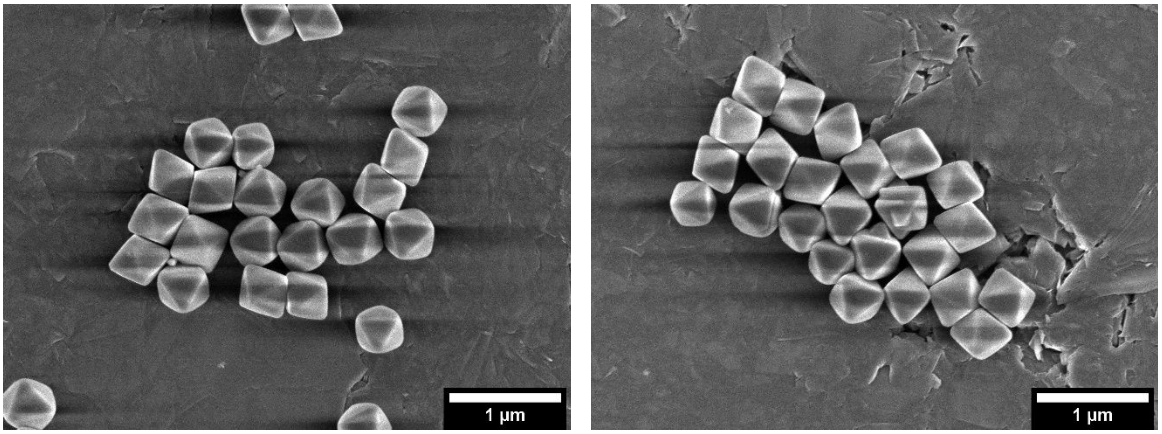

Figure S33. SEM images of UiO-66-Br nanoparticles before (left) and after (right) PSE with H_2BDC. The magnification of the SEM images is 25000x.

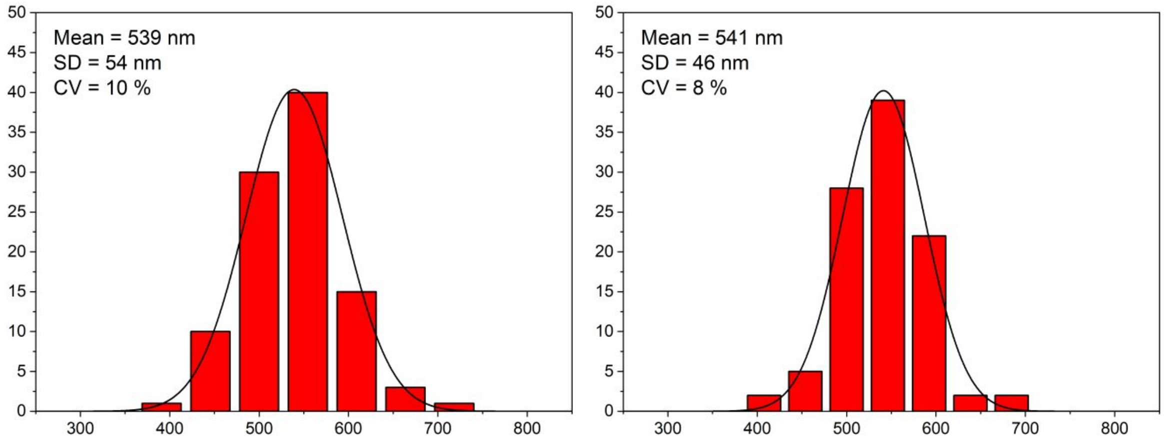

Figure S34. Particle size distribution from SEM images (100 particles measured each) of UiO-66-Br nanoparticles before (left) and after (right) the PSE with H_2BDC.

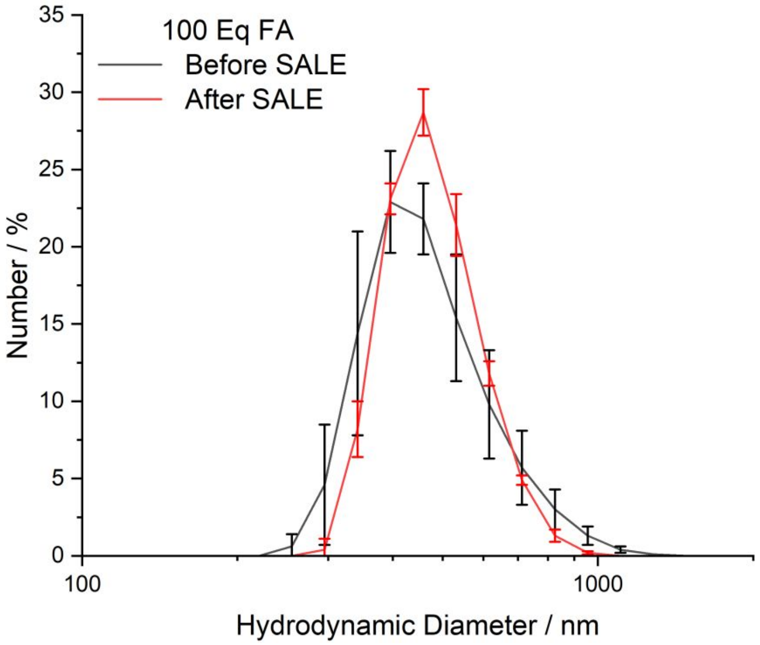

Figure S35. DLS measurements of UiO-66-Br nanoparticles before (black line) and after (red line) PSE with H_2BDC. The values given are the average of three measurements.

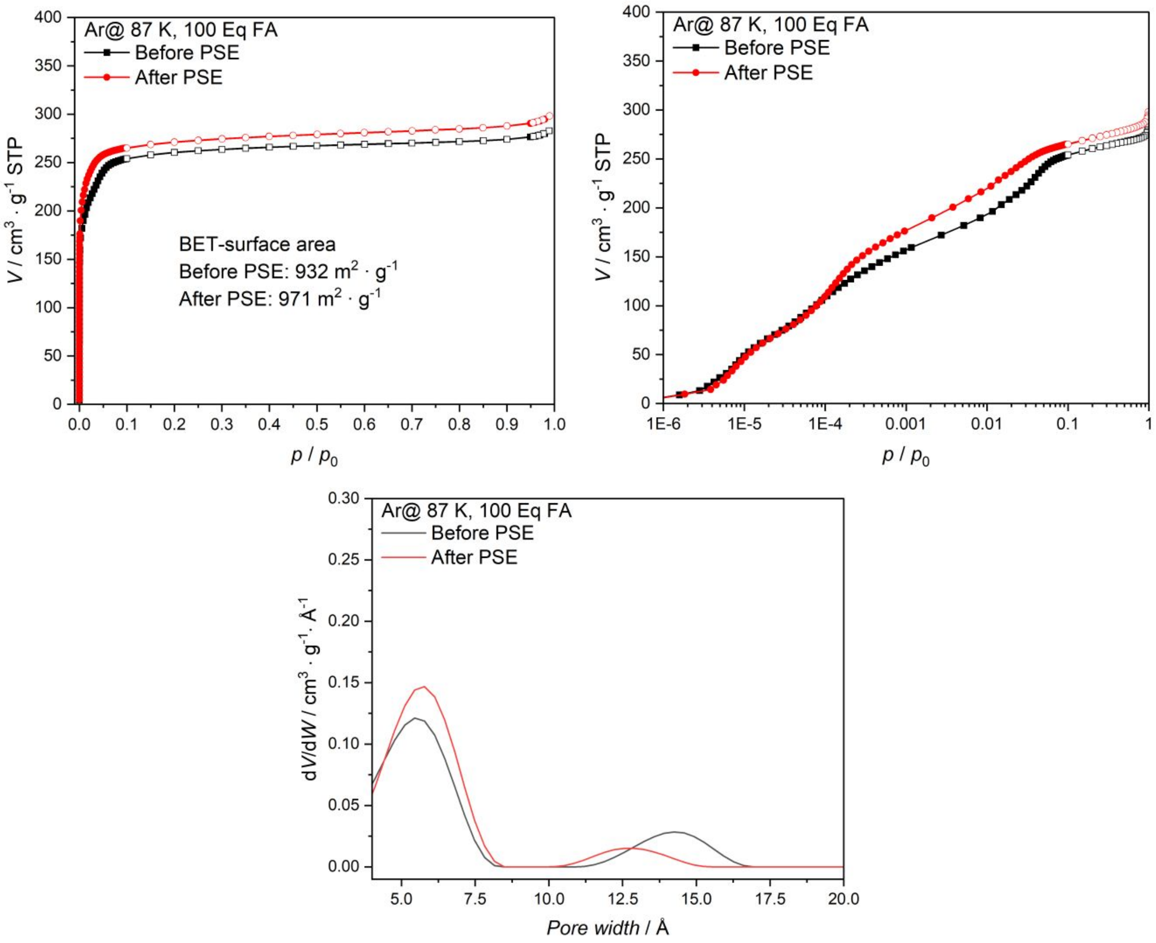

Figure S36. Argon-sorption measurements at 87 K on UiO-66-Br nanoparticles after PSE with H_2BDC. Top left: Argon-sorption isotherms plotted linear, top right: Argon-sorption isotherms plotted on a logarithmic scale. Bottom: Pore size distribution calculated from argon-sorption isotherms.

STEM-EDX measurement on UiO-66-Br_H core shell nanoparticles

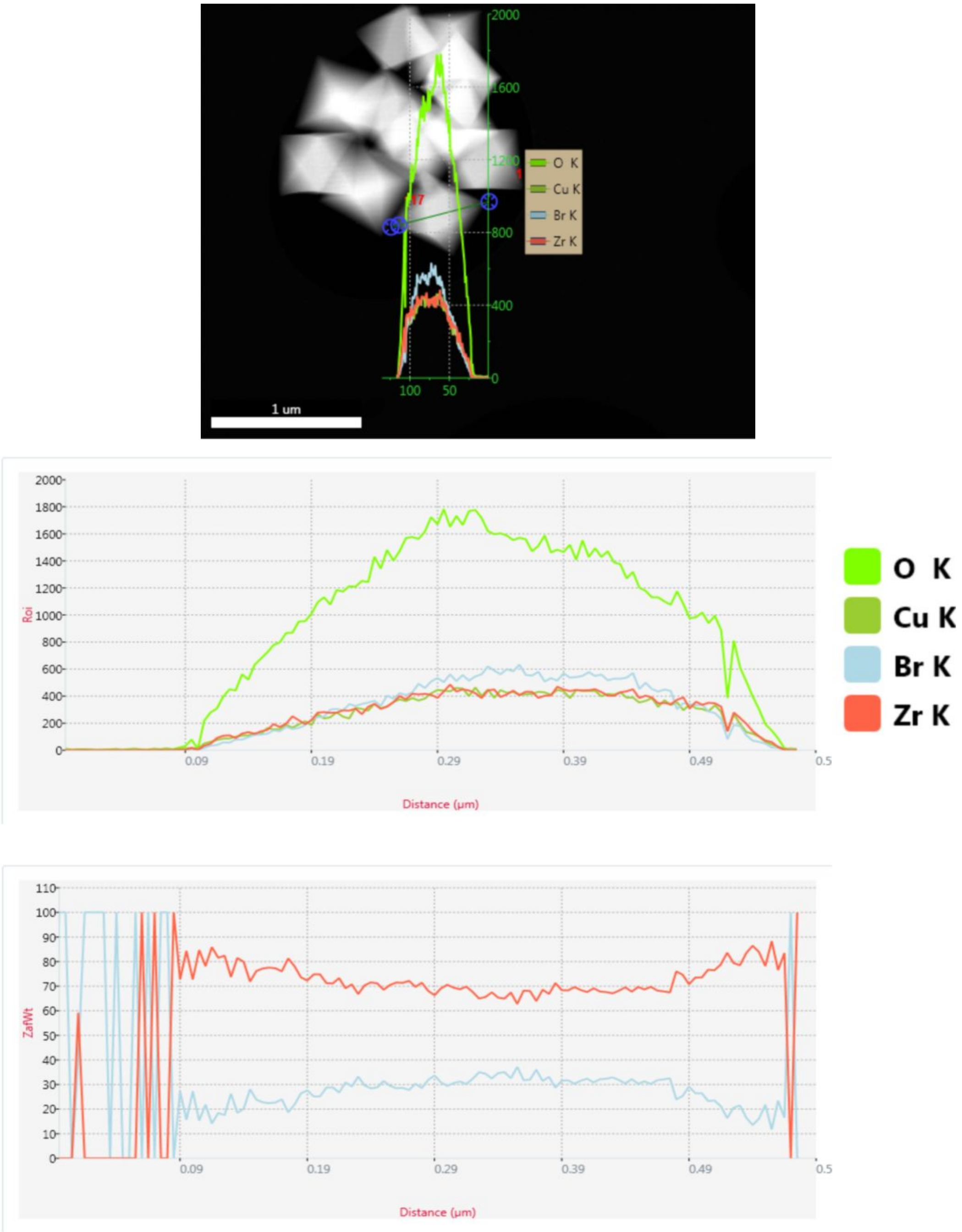

Figure S37. STEM-EDX measurement on UiO-66-Br nanoparticles after the PSE with H_2BDC. Top: TEM image with EDX line scan. Middle: STEM-EDX line scan showing all detected elements despite carbon (always detected from TEM grid), Cu could also be detected because of the TEM grid. Bottom: Normalized intensity comparing Zr to Br. The particle ends at the points (beginning and end of the line) where there is a strong signal-to-noise ratio.
